# Using Land Runoff To Survey the Distribution and Genetic Diversity of Burkholderia pseudomallei Strains in Vientiane, Laos

**DOI:** 10.1128/AEM.02112-20

**Published:** 2021-01-29

**Authors:** Audrey Rachlin, Manophab Luangraj, Mirjam Kaestli, Sayaphet Rattanavong, Phonelavanh Phoumin, Jessica R. Webb, Mark Mayo, Bart J. Currie, David A. B. Dance

**Affiliations:** a Global and Tropical Health Division, Menzies School of Health Research, Charles Darwin University, Darwin, Northern Territory, Australia; b Lao-Oxford-Mahosot Hospital-Wellcome Trust Research Unit, Microbiology Laboratory, Mahosot Hospital, Vientiane, Laos; c Research Institute for the Environment and Livelihoods, Charles Darwin University, Darwin, Northern Territory, Australia; d Department of Infectious Diseases, Royal Darwin Hospital, Darwin, Northern Territory, Australia; e Northern Territory Medical Program, Royal Darwin Hospital, Darwin, Northern Territory, Australia; f Centre for Tropical Medicine and Global Health, Nuffield Department of Clinical Medicine, Old Road Campus, University of Oxford, Oxford, United Kingdom; g Faculty of Infectious and Tropical Diseases, London School of Hygiene and Tropical Medicine, London, United Kingdom; University of Michigan—Ann Arbor

**Keywords:** *B. pseudomallei*, land runoff, Laos, melioidosis, phylogeography, whole-genome sequencing

## Abstract

Melioidosis is a disease of significant public health importance that is being increasingly recognized globally. The majority of cases arise through direct percutaneous exposure to its etiological agent, Burkholderia pseudomallei. In the Lao People’s Democratic Republic (Laos), the presence and environmental distribution of B. pseudomallei are not well characterized, though recent epidemiological surveys of the bacterium have indicated that B. pseudomallei is widespread throughout the environment in the center and south of the country and that rivers can act as carriers and potential sentinels for the bacterium. The spatial and genetic distribution of B. pseudomallei strains within Vientiane Capital, where the majority of cases diagnosed to date have originated, remains an important knowledge gap. We sampled surface runoff from drain catchment areas throughout urban Vientiane to determine the presence and local population structure of the bacterium. B. pseudomallei was detected in drainage areas throughout the capital, indicating that it is widespread in the environment and that exposure rates in urban Vientiane are likely higher than previously thought. Whole-genome comparative analysis demonstrated that Lao B. pseudomallei isolates are highly genetically diverse, suggesting that the bacterium is well established and not a recent introduction. Despite the wide genome diversity, one environmental survey isolate was highly genetically related to a Lao melioidosis patient isolate collected 13 years prior to the study. Knowledge gained from this study will augment understanding of B. pseudomallei phylogeography in Asia and enhance public health awareness and future implementation of infection control measures within Laos.

**IMPORTANCE** The environmental bacterium B. pseudomallei is the etiological agent of melioidosis, a tropical disease with one model estimating a global annual incidence of 165,000 cases and 89,000 deaths. In the Lao People’s Democratic Republic (Laos), the environmental distribution and population structure of B. pseudomallei remain relatively undefined, particularly in Vientiane Capital, where most diagnosed cases have originated. We used surface runoff as a proxy for B. pseudomallei dispersal in the environment and performed whole-genome sequencing (WGS) to examine the local population structure. Our data confirmed that B. pseudomallei is widespread throughout Vientiane and that surface runoff might be useful for future environmental monitoring of the bacterium. B. pseudomallei isolates were also highly genetically diverse, suggesting that the bacterium is well established and endemic in Laos. These findings can be used to improve awareness of B. pseudomallei in the Lao environment and demonstrate the epidemiological and phylogeographical insights that can be gained from WGS.

## Abstract (precis)

The environmental bacterium B. pseudomallei is the etiological agent of melioidosis, a tropical disease with one model estimating a global annual incidence of 165,000 cases and 89,000 deaths. In the Lao People’s Democratic Republic (Laos), the environmental distribution and population structure of B. pseudomallei remain relatively undefined, particularly in Vientiane Capital, where most diagnosed cases have originated.

## INTRODUCTION

Melioidosis is a serious disease of humans and animals caused by the environmental Gram-negative sapronotic bacterium Burkholderia pseudomallei. Infection results from inoculation, inhalation, or ingestion of B. pseudomallei and is fatal in 10 to 40% of human cases (1, 2). Melioidosis was first reported for a patient in the Lao People’s Democratic Republic (Laos) in 1999 (3), and since that case, 1,690 culture-positive Lao melioidosis patients have been confirmed by the Microbiology Laboratory of Mahosot Hospital in Vientiane as part of an ongoing prospective study (unpublished data) (4). While the infection has now been established as being highly endemic in Laos, the true burden of melioidosis and the environmental distribution of B. pseudomallei remain relatively undefined.

B. pseudomallei is recognized as being spatially heterogeneously distributed on both broad and localized geographical scales in soil (5). This restricted geographical distribution has resulted in a robust global biogeographic structure, with genetic populations being highly spatially clustered in the environment despite high levels of gene recombination and sequence type (ST) diversity (6–8). Whole-genome sequencing (WGS) has facilitated the examination of genetic populations of the bacterium on a fine scale (6, 9–11) and has revealed large-scale geographical partitioning between Australian and Southeast Asian isolates as well as highly localized genetic spatial clustering (6, 8, 12, 13).

The spatial heterogeneity of isolates means that the use of random soil sampling to establish the bacterial presence in a region can often be indeterminate and imprecise (5, 14). It has been suggested that the identification of new environments where melioidosis is endemic may be effectively achieved by analyzing catchment points along the water column, including groundwater and surface runoff areas (14–16). Since stormwater is known to capture and leach what is in the land, including particulates, contaminants, and bacteria, it is thought that it may provide a good indication of B. pseudomallei distribution within a catchment, as the bacterium is able to disperse along the water table and via drainage lines (17, 18). Moreover, direct sampling of the water column and surface water can also provide an indication of the associations between environmental physicochemical factors within a catchment (14).

In Laos, recent surveys have shown that water may be a significant reservoir and transport vehicle for B. pseudomallei (14, 15, 19). In one recently published study, the bacterium was isolated in 57% of samples collected during the rainy season from the Mekong River and its tributaries in the center and south of the country (15), and it has also been detected in surface water and catchment areas in Salavan Province in the south of the country (14, 19).

High levels of B. pseudomallei have also been isolated in groundwater and groundwater seeps in both Townsville (16) and Darwin (13), Australia, and groundwater isolates have been linked to clinical isolates using molecular typing (16). However, the extent to which groundwater and seasonal runoff are contaminated with B. pseudomallei and might contribute to melioidosis in Laos has not yet been assessed, and there is limited knowledge about the ST distribution and genomic diversity of isolates. Moreover, very few environmental surveys of the bacterium have been undertaken in Vientiane Capital, where over 10% of the Lao population currently resides and where just over half (54.6%) of more than 1,359 culture-confirmed Lao melioidosis patients between 1999 and 2017 reported living (4). As most of the culture-confirmed melioidosis patients from Mahosot Hospital reside in Vientiane, we surveyed surface runoff at drainage catchment areas across the city. WGS and large-scale comparative genomics were performed on cultured isolates to examine the phylogenetic relatedness and population structure of B. pseudomallei in Laos to improve knowledge of genotype diversity. This has important global relevance given the substantial numbers of undetected melioidosis cases and deaths predicted to occur annually throughout Laos and Southeast Asia (20).

## RESULTS

### Detection of B. pseudomallei at drain sites.

Results of culture and direct detection of B. pseudomallei in water samples are shown in Table 1. B. pseudomallei was detected in water collected at 62.5% (25/40) of sites by standard culture and/or direct PCR extraction techniques. At only two water-positive drain sites (7.7% [2/26]) did all three water samples test positive for the bacterium. B. pseudomallei was detected more frequently using molecular detection techniques than by conventional culture for water samples (Table 1).

**TABLE 1:** B. pseudomallei positive sites and samples based on different detection techniques

Method of detection	% (no./total) for:	
	Sites	Water samples
Culture positive	22.5 (7/40)	11.7 (14/120)
Direct DNA extraction PCR positive	45.0 (18/40)	21.7 (26/120)
Direct DNA extraction PCR negative, culture positive	10 (4/40)	5 (6/120)
Both methods positive	12.5 (5/40)	6.7 (8/120)
Total	62.5 (25/40)	33.3 (40/120)

**FIG 1:**
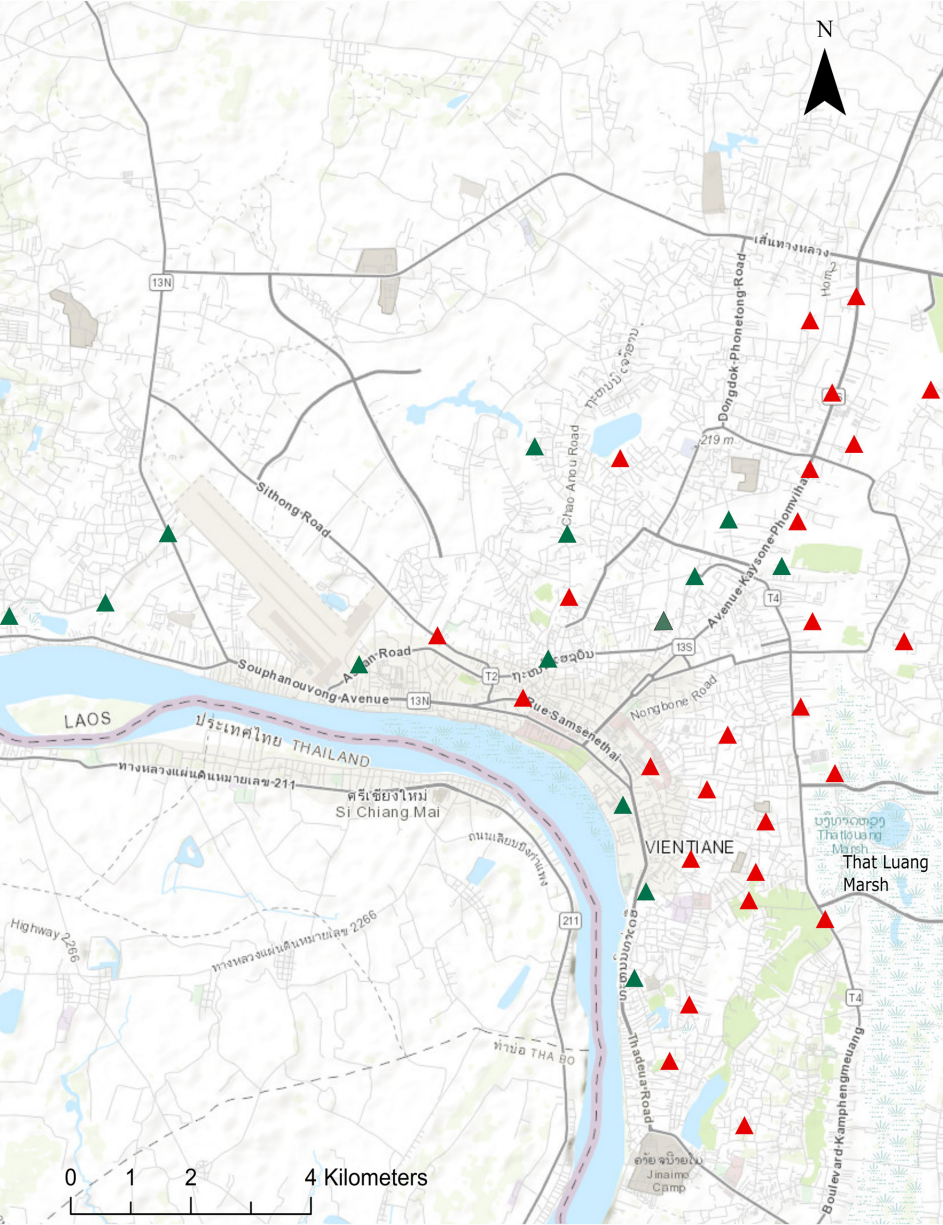
Sampling site locations across urban Vientiane Capital. Sites where B. pseudomallei was detected by culture and/or direct detection in water are indicated by red triangles. Negative sites (green triangles) are those where B. pseudomallei was not identified in water by either detection method. The base map is from ArcGIS/Esri (sources: Esri, HERE, Garmin, Intermap, increment P Corp., GEBCO, USGS, FAO, NPS, NRCAN, GeoBase, IGN, Kadaster NL, Ordnance Survey, Esri Japan, METI, Esri China [Hong Kong], OpenStreetMap contributors, and the GIS user community).

Positive sites were scattered throughout the city, though some clustering was observed around the That Luang Marsh area in Xaysetha and Sisattanak districts. Global Moran’s I and corresponding Z-score also suggested that there was positive spatial autocorrelation between B. pseudomallei-positive sites (I = 0.31, Z = 2.21, *P* < 0.05). Fewer positive sites were observed in west and northwest areas of the city (Fig. 1).

Of the 1,690 patients identified in the prospective Lao melioidosis study between October 1999 and September 2020, 10 had incomplete residence data. Of the remaining 1,680 patients, 55.5% (933/1,680) reported having a residential address in Vientiane Capital, of whom 63.5% (592/933) resided within the five study districts. Cases were most frequently reported from Xaythany (44.8% [265/592]) and Sikhottabong (24.3% [144/592]) districts, with no obvious overrepresentation in the That Luang Marsh area, where spatial clustering of positive environmental samples was found. In view of the inherent biases in the selection of both sampling sites and patient residence, however, formal analysis of associations was not attempted.

### Physicochemical parameters.

Characteristics of physicochemical water parameters from sites (turbidity [formazin nephelometric units {FNU}], temperature, total dissolved solids [TDS], nitrate, acidity [pH], salinity [electrical conductivity], dissolved oxygen [DO], redox potential [ORP], coliform and Escherichia coli counts, drain type, and district where located) are shown in the supplemental material (Table S2). Conductivity differed considerably between samples (49 to 908 μS/cm), as did turbidity (1.1 to 851 FNU) and TDS (41 to 377 ppm). Nitrate content was also variable (8 to 28 mg/liter), as were E. coli and coliform counts (both 0 to >250 CFU/ml) and redox potential (−150.8 to 192.1 mV). Temperature ranged between 26.3°C and 33.2°C. In contrast, pH varied only by approximately 2 units (6.36 to 8.45) and DO fluctuated between 0 and 4.4 mg/liter.

### Physicochemical associations with B. pseudomallei occurrence in drain water.

For the water samples, there was a positive association between the presence of B. pseudomallei in water with turbidity, TDS, and unlined drain sites, as well as slightly cooler temperature (univariable generalized estimating equation [GEE] models, *P* < 0.05 for all) (Table 2 and Fig. S1). B. pseudomallei was also less likely to be isolated from the Sikhottabong (*P* = 0.049) and Chanthabuly (*P* = 0.017) districts than from the Xanthany district (Table 2). There was no association observed between B. pseudomallei and additional variables measured as part of the study (Fig. S2).

**TABLE 2:** Multivariable GEE analysis of water parameters associated with the presence of B. pseudomallei in Vientiane drains

Variable	Positive	Negative	Multivariable GEE model OR (95% CI) and *P* value^*a*^
Turbidity (FNU; log transformed in GEE), median (range)	79.2 (13.1–851)	32.7 (1.1–461)	2.42 (1.31–4.5), 0.005**
TDS (ppm), median (range)	192 (103–377)	152 (41–304)	1.01 (1.01–1.02), 0.006**
Drain lining, % (no./total)			
Cement lined	2.5 (3/120)	20 (24/120)	0.14 (0.02–0.88), 0.036*
Unlined	30.8 (37/120)	46.7 (56/120)	Reference level
District, % (no./total)			
Sisattanak	15 (18/120)	15 (18/120)	0.9 (0.32–2.61), 0.87
Xaysetha	7.5 (9/120)	12.5 (15/120)	0.43 (0.15–1.26), 0.13
Xaythany	5.8 (7/120)	6.7 (8/120)	Reference level
Sikhottabong	1.7 (2/120)	15.8 (19/120)	0.09 (0.009–0.72), 0.02*
Chanthabuly	3.4 (4/120)	16.7 (20/120)	0.01 (0.08–1.57), 0.17

a Asterisks indicate levels of significance as follows: *, *P* < 0.05, and **, *P* < 0.01.

**FIG 2:**
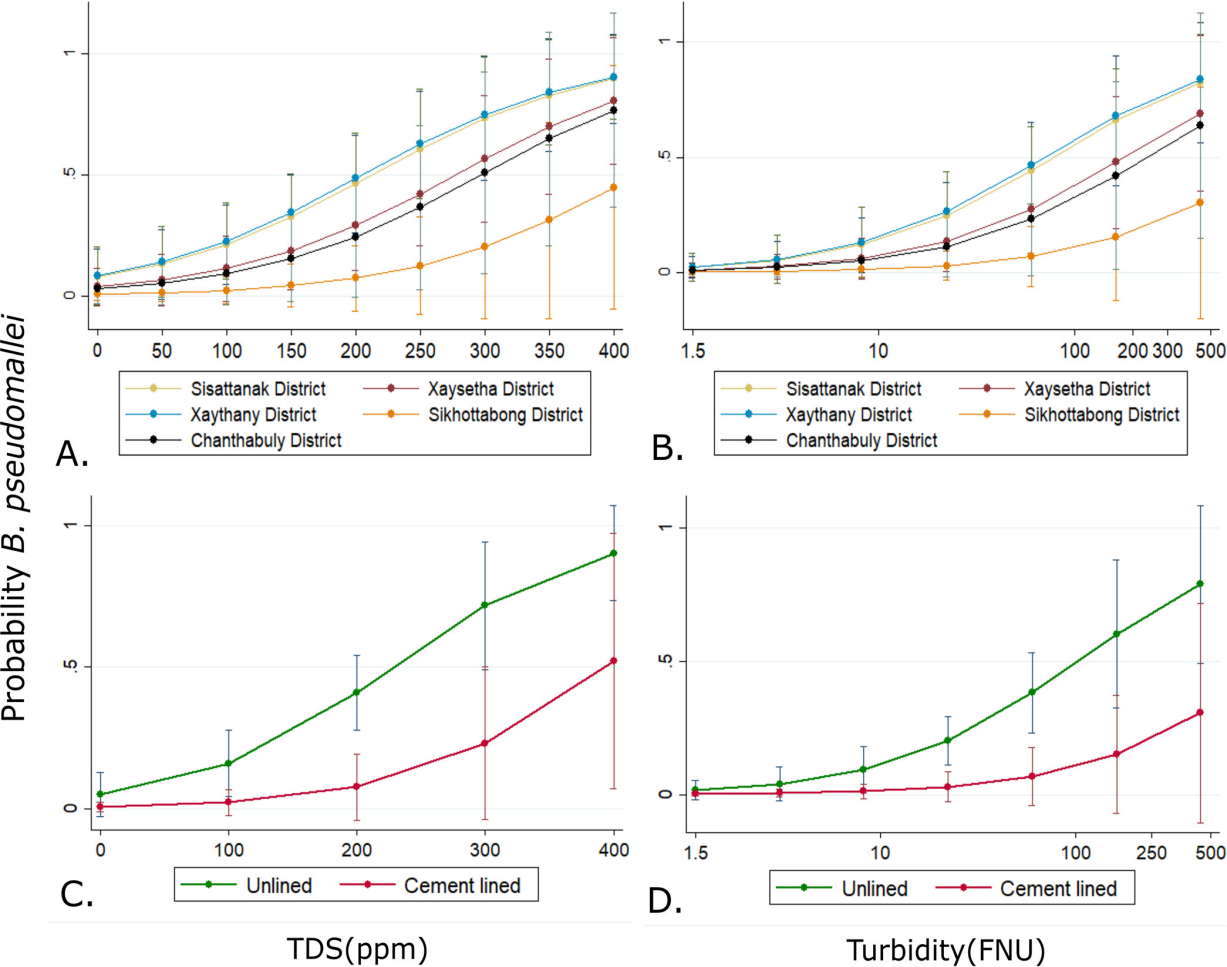
Generalized estimated equation (GEE) margins plots of adjusted predicted probabilities of B. pseudomallei occurrence with all other variables in the model held constant. Shown are predicted probabilities as a function of increased water TDS (A) in each of the districts surveyed and turbidity (B) or probabilities in unlined or cement-lined drains based on TDS (C) or turbidity of water sample (D). Bars indicate 95% confidence intervals (CIs).

A multivariable GEE model showed that B. pseudomallei was negatively associated with drains that were lined with cement rather than those that were sediment lined and was less likely to be detected in Sikhottabong than in the Sisattanak, Chanthabuly, Xaythany, and Xaysetha districts. Water samples higher in turbidity and total dissolved solids were also positively associated with the detection of B. pseudomallei. However, after accounting for district, turbidity, and TDS, water temperature was no longer significantly associated with the presence of B. pseudomallei in the multivariable GEE model (Table 2 and Fig. 2).

### Population structure of B. pseudomallei in Laos.

From the 25 B. pseudomallei Vientiane soil and water isolates selected for WGS, we identified 10 distinct multilocus sequence type (MLST) genotypes (Table 3). ST-507 was the most frequently observed molecular type (*n* = 11), followed by ST-376 (*n* = 3). ST-1792 was the only novel ST identified. There were two STs (ST-70 and ST-654) that were identified in soil only, while seven STs were isolated in water but not soil. Only one ST, ST-507, was isolated from both sample types.

**TABLE 3:** Vientiane B. pseudomallei study isolates included in whole-genome comparative analysis

Isolate	Sample type	Vientiane village and district	ST
MSHR12071	Water	Phonphapao, Sisattanak	46
MSHR11998	Water	Phonphapao, Sisattanak	52
MSHR12336	Soil	Phonthan Neua, Xaysetha	70
MSHR12338	Soil	Phonthan Neua, Xaysetha	70
MSHR12048	Water	Phonphapao, Sisattanak	203
MSHR12122	Water	Tonphanthong, Sisattanak	203
MSHR11848	Water	Saphanthong, Sisattanak	368
MSHR12054	Water	Saphanthong, Sisattanak	368
MSHR11846	Water	Tonphanthong, Sisattanak	376
MSHR12046	Water	Phonphapao, Sisattanak	376
MSHR12097	Water	Tonphanthong, Sisattanak	376
MSHR11836	Water	Phonphapao, Sisattanak	507
MSHR11855	Water	Saphanthong Tai, Sisattanak	507
MSHR11859	Water	Saphanthong Tai, Sisattanak	507
MSHR11966	Water	Nongchan, Chanthabuly	507
MSHR12012	Water	Saphanthong Tai, Sisattanak	507
MSHR12020	Water	Sengsavanh, Sikhottabong	507
MSHR12061	Water	Saphanthong Tai, Sisattanak	507
MSHR12077	Water	Sengsavanh, Sikhottabong	507
MSHR12103	Water	Nongchan, Chanthabuly	507
MSHR12347	Soil	Sybounheung, Xathany	507
MSHR12414	Soil	Sybounheung, Xathany	507
MSHR12000	Water	Phonphapao, Sisattanak	535
MSHR12369	Soil	Phonthan Neua, Xaysetha	654
MSHR12059	Water	Sapangmor, Sisattanak	1792^*a*^

a Novel ST.

All nine nonnovel STs had been recorded in at least one nearby Asian country. These countries included Thailand, Cambodia, Vietnam, and China as well as Bangladesh, Malaysia, Singapore, and Indonesia. Three of the nine environmental STs, ST-70, ST-376, and ST-507, had also been identified in Lao melioidosis patients previously (https://pubmlst.org/bpseudomallei/).

**FIG 3:**
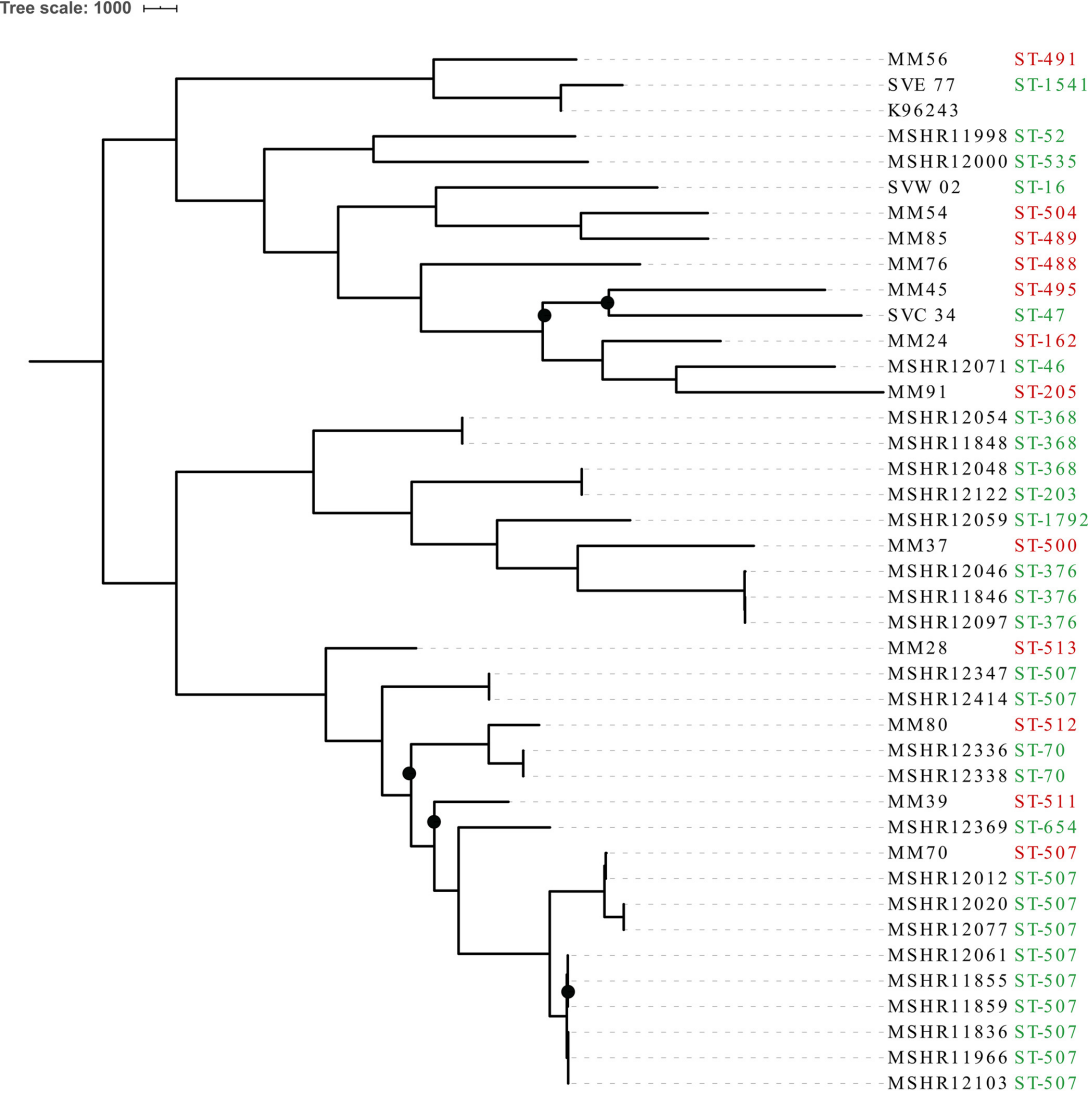
Midpoint rooted maximum parsimony phylogeny of 40 Lao B. pseudomallei isolates based on 56,532 core genome SNPs and indels. Black circles indicate bootstrap values of <80.

A phylogenetic tree was constructed using the 25 study isolates as well as an additional 15 publicly available clinical and environmental Lao genomes, comprising 24 individual STs (Fig. 3). Concurrent with the high degree of ST diversity, comparative genomics demonstrated that the Lao isolates were highly genetically diverse, with 56,532 orthologous core genome single nucleotide polymorphisms (SNPs) and indels detected among the 40 isolates. Two distinct clades separated by 4,460 SNPs/indels were also identified, comprising 13 and 11 ST types, respectively. Isolates did not group by whether they were clinical or environmental, and while most isolates grouped by ST, two ST-507 isolates (MSHR12347 and MSHR12414) did not cluster with the other ST-507 genomes. Both isolates, which were recovered from the same soil sample and differed from one another by only three SNPs/indels, were separated from the other nine ST-507 isolates by more than 8,355 SNPs/indel variants. This distance is consistent with previously reported occurrences of B. pseudomallei MLST homoplasy (21, 22) and likely represents homoplasy occurring within Laos. Additionally, phylogenetic analysis demonstrated that one publicly available ST-507 Lao melioidosis patient isolate, MM70, was closely related to a survey water isolate. The water sample isolate (MSHR12012), which was recovered from Saphanthong Tai Village in eastern Vientiane City, differed from the clinical patient genome by only 66 SNP/indel variants (Fig. 3). This is despite MM70 having been isolated in 2005 from a Lao melioidosis patient with a residential address in Bolikhamxai Province, approximately 150 km northeast of Vientiane City.

### Comparative analysis demonstrates that B. pseudomallei is diverse and well established in Laos.

**FIG 4:**
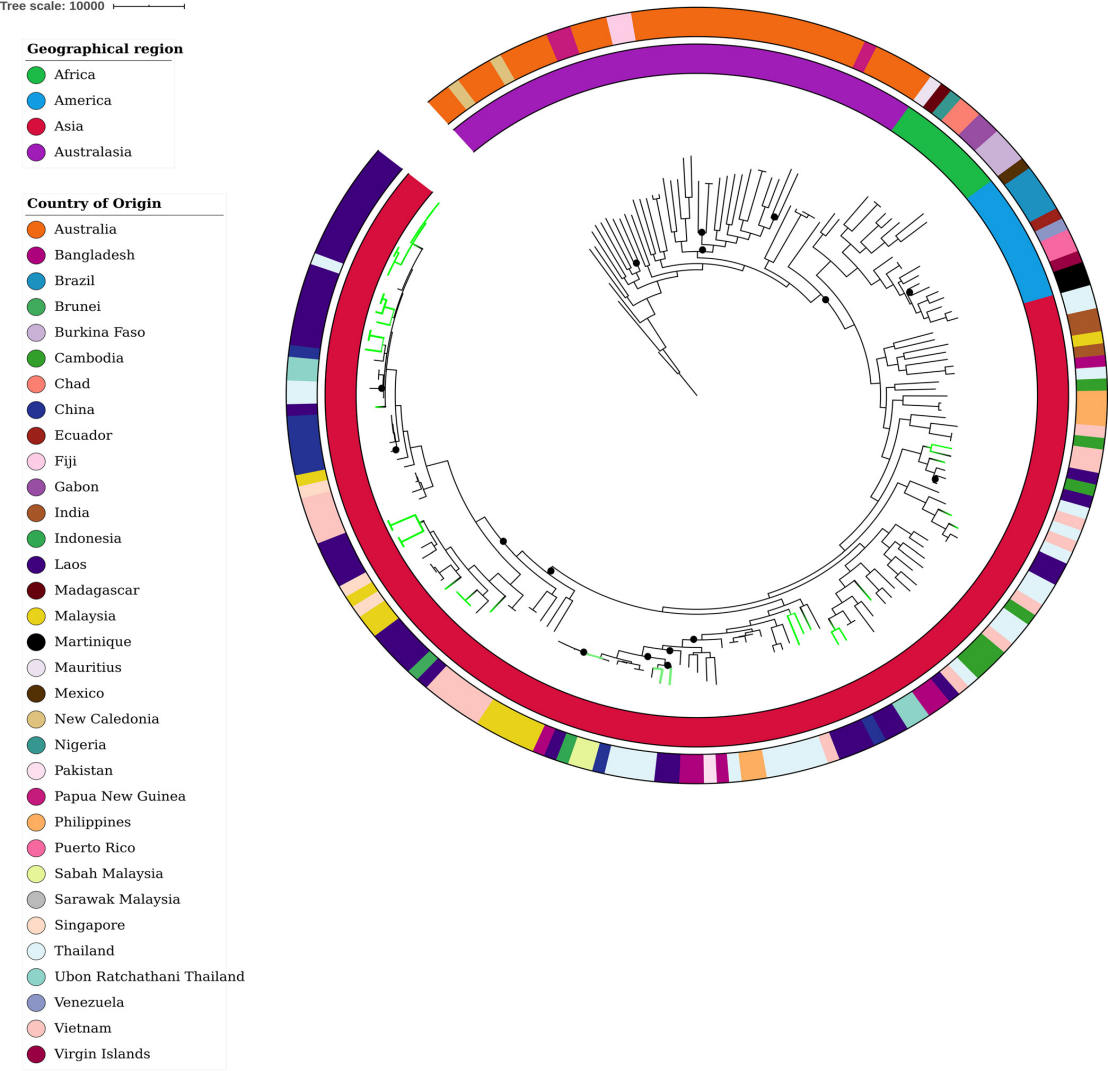
Maximum-parsimony phylogeny of B. pseudomallei from Laos (*n* = 40, green branches) with a global set of genomes (*n* = 159) based on 168,934 core genome SNPs and indels. The closed Thai isolate K96243 was used as the reference strain and the tree was rooted at MSHR0668, the most ancestral B. pseudomallei strain identified in a previous study (31). Black circles indicate bootstrap values <80.

Whole-genome comparison of the 40 Lao B. pseudomallei genomes with an additional 159 global isolates identified 168,934 core SNPs and indel variants. Lao isolates (green branches in Fig. 4) clustered in multiple distinct groups within the Asian clade, with some appearing to have arisen earlier based on their proximity to the more ancestral Australian strains. These strains (ST-52, ST-491, and ST-535) were more genetically diverse and had longer branches than strains residing at the end of the global phylogeny. ST-507 isolates appeared to be the least diverse and most recently evolved strain we detected from Laos.

The 40 Lao isolates also shared nodes and clustered with strains from multiple nearby Asian countries, indicating distinct recent common ancestors. These included isolates from Thailand, China, Vietnam, Cambodia, Singapore, Bangladesh, Malaysia, and Indonesia (Fig. 4). ST-507 strains grouped closely and shared nodes with Thai and Chinese isolates, in some instances differing by fewer than 1,100 SNPs and indels. One Lao isolate (MSHR12071; ST-46) was separated by strains from Bangladesh and Malaysia by 233 and 327 SNPs/indels, respectively.

## DISCUSSION

Given the spatial heterogeneity of B. pseudomallei distribution in soil, unknown regions where melioidosis is endemic may be effectively identified through the analysis of integrated catchment points along a water column (5, 14). We investigated the presence and genetic diversity of B. pseudomallei in urban Vientiane, Laos, by assessing surface runoff and drainage catchment points throughout the city center. B. pseudomallei was detected at the majority of sites surveyed across all districts, indicating that it is well established there and that surface runoff, particularly during periods of increased rainfall, might be useful for future environmental monitoring of the bacterium. Whole-genome comparison of isolates also demonstrated that Lao B. pseudomallei strains are highly genetically diverse, suggesting that introduction to Laos has not been a recent occurrence and that the bacterium has long been endemic there.

During periods of heavy rainfall and increased surface discharge, B. pseudomallei is likely washed out of the soil and channelled into drainage areas along with other eroded particulate matter. Consequently, turbidity and increased suspended solids are thought to be important correlates of the presence of B. pseudomallei in water, since bacteria tend to attach to soil and sediment particles rather than exist in their free state (17). This association has been observed previously with fecal indicator bacteria after heavy-rainfall events (14, 15, 17). Accordingly, we identified a positive association between B. pseudomallei and turbid, particle-rich water, as has been observed previously with B. pseudomallei isolated from rural domestic water supplies in Northern Australia (23) and in rivers and tributaries throughout southern Laos (14, 15). Additionally, the presence of the bacterium was also found to be associated with sediment-laden unlined drains, suggesting that sediment might act as an additional reservoir for the pathogen and also supporting a link between bank erosion and B. pseudomallei particle-bound transport (17). This finding also supports the lack of correlation we detected between B. pseudomallei-positive water samples and the presence of fecal coliforms. Abundant enteric microorganisms have been shown to outcompete B. pseudomallei in the environment previously, and fewer coliforms in a sample may enable the growth of B. pseudomallei (17). The lack of correlation identified may reflect variations in the origins of increased turbidity, such as soil runoff rather than fecal contamination.

Land use can play an integral role in the transfer of bacteria through soils to downstream aquatic systems and catchment areas (14, 24, 25). As Vientiane Capital continues to develop and expand, changes in land use may ultimately lead to increased soil erosion and runoff. This could potentially affect the distribution and dispersal of the bacterium there, particularly during periods of heavy rainfall (14, 18). Thus, the potential for increased rates of B. pseudomallei transmission and its propagation to uncontaminated areas should be considered as the city continues to grow.

Moreover, though we detected B. pseudomallei in all districts surveyed as part of the investigation, there was evidence for spatial clustering of the bacterium across the city. Despite the small geographical area surveyed, B. pseudomallei was detected at a lower rate in west and northwest areas of Vientiane, again indicative of the heterogeneous nature of the bacterium in the environment. Additionally, some clustering of positive sites was also observed around That Luang Marsh, located on the eastern edge of the city. The marsh, which is the largest wetland area in Vientiane Municipality, has been designed to collect and treat runoff and drainage water from Vientiane and surrounding areas and also provides local irrigation to farmers (26, 27). The increased degree of runoff could indicate why we observed some clustering of positive sites in this region and why B. pseudomallei was detected at a lower rate in the western areas of the city. However, bias caused by nonrandom sampling due to accessibility and site approval from local authorities should not be discounted as possible study limitations.

For water samples, we applied two separate detection methods including direct type three secretion system 1 (TTS1) quantitative PCR (qPCR) on DNA extracted postenrichment. This has been demonstrated to be a more sensitive technique for the detection of B. pseudomallei in the environment than standard bacterial culture. Despite this, 6 of the 120 water samples were detected by culture-only methods and were negative by qPCR post-direct DNA extraction, confirming that no single detection technique is 100% sensitive (28–30). In contrast, soil samples were processed using less sensitive culture methods than those that are usually recommended, and confirmation of B. pseudomallei was performed only on small quantities of shipped bacterial cultures due to constraints of project time and budget. While water samples were also filtered and processed promptly after collection, soils were stored for several months before being cultured and shipped back to Darwin, potentially decreasing the viable bacterial count to below the limit of detection. Tropical soils have been demonstrated to be the natural environmental reservoir for B. pseudomallei detected in rivers and groundwater, with the bacterium leached out of the soil along with eroded particulate matter during periods of heavy rainfall (14, 25). Consequently, it is likely that the pathogen was present in many of the soils collected in water-positive survey sites but was not detected by our collection and processing methods. Future comparisons between the roles and links of water and soil will require more intensive soil sampling and on-site processing and analysis.

Although B. pseudomallei has one of the most highly recombinogenic genomes of any bacteria, certain features of its biology mean that reliable inferences about geographic origin and population structure can still be made, particularly when high-resolution WGS data are used (31). In 2009, Pearson and colleagues were the first to hypothesize an Australian origin for B. pseudomallei. Combining WGS, Bayesian inference, and molecular clock estimates, they predicted that B. pseudomallei moved into Southeast Asia during the last glacial period (16 to 225 thousand years ago), when the Sahul and Sunda land masses were in close proximity due to low sea levels (6). Studies across larger more diverse sets of data have supported this hypothesis, and it has recently been shown that there have been several successive B. pseudomallei reintroductory events within Southeast Asia. This was particularly evident among countries bordering the Mekong River and Malay Peninsula, where there was a high degree of genetic relatedness and shared ancestry among isolates (8). Given that Laos has geographical borders with five Southeast Asian countries and the Mekong River runs along its western boundary, the extent of ST diversity and genetic relatedness we observed among B. pseudomallei isolates from Laos and those from neighboring countries was unsurprising. Collectively, the diversity and divergence of isolates within Laos suggest that the original introduction of B. pseudomallei did not happen recently and the disease has long been endemic there.

Moreover, Lao genomes clustered in different clades within our global phylogeny. This could indicate that B. pseudomallei was introduced to Laos on multiple separate occasions, with isolates having distinct recent common ancestors. These repeated introductory events and the subsequent dispersal of B. pseudomallei within Laos are likely multifaceted. Severe weather and flooding during the monsoonal season have probably played an important role, as has its close proximity to neighboring countries, which would enable transmission by both humans and animals. More environmental sampling and sequencing of isolates throughout Laos will be necessary to elucidate the time frame in which these introductory events may have occurred and further explore the phylogeographic relatedness with other Southeast Asian isolates.

Our results also revealed that B. pseudomallei isolates from Laos are highly genetically diverse, with 24 STs and 56,532 orthologous core SNPs and indels identified among the 40 sequenced isolates. Likewise, we identified 10 individual STs among the 25 environmental survey isolates sent for WGS within the small (100 km^2^) study radius. Despite the overall degree of genetic diversity, whole-genome comparison identified two highly related ST-507 isolates: a Lao clinical isolate from 2005 and a Vientiane survey water isolate from 2018 collected from Saphanthong Tai Village. Despite being isolated 13 years apart, the genomes were separated by only 66 core SNPs and indels. Additionally, the patient’s residential address was approximately 150 km northeast of the source of the environmental isolate. Genetic populations of B. pseudomallei have been demonstrated to spatially cluster in the environment on a highly localized scale despite frequent opportunities to spread within the water table, via agricultural and migratory animals, or in transported soil (7, 16, 32, 33). Genetic clustering has also been shown to match the spatial distribution of clinical cases previously (13). This might indicate that the patient did not acquire their infection at their residential address but closer to the location where the survey isolate was collected in Vientiane, although more detailed clinical epidemiological data would be required to determine this. Alternatively, this finding could suggest that ST-507 is comparatively widespread throughout central Laos and that there is limited intra-ST-507 diversity. This is supported by results from our global phylogeny, which demonstrated that ST-507 is a more recently evolved and less genetically diverse B. pseudomallei sequence type than many others. Additional sampling and WGS of clinical and environmental isolates from Laos are needed to further examine this, since relatively few Lao isolates have had MLST or WGS completed.

Previous environmental surveys undertaken in Laos have shown that B. pseudomallei is widespread throughout the central and southern regions of the country, with high concentrations of the bacterium identified in rice paddies in rural Vientiane Province (4, 34–36). Results from this study indicate that B. pseudomallei is also widespread in the environment throughout urban Vientiane, where more than 10% of the Lao population currently resides and where over half of the individuals diagnosed with melioidosis at Mahosot Hospital lived (4). While the true distribution and epidemiology of melioidosis are still not well characterized in Laos, our results indicate that infection from contact with the environment is a significant risk in urban Vientiane, with drains and surface runoff being potential sources in addition to more conventional sources such as agricultural land. The rate of B. pseudomallei detection in drain water across the study area also corresponded with the high proportion of Lao melioidosis patients reporting residential addresses within the five urban districts, comprising a third (35.2% [592/1,680]) of all cases confirmed at Mahosot Hospital. Despite this, patient addresses did not appear to cluster around That Luang Marsh like the positive environmental sites. This finding may reflect the nonrandom aspects of environmental sampling in addition to population density, underlying risk factors, access to health care in these areas, and the fact that not all patients will have been infected at their residential address.

## MATERIALS AND METHODS

### Study sites and sample collection.

**FIG 5:**
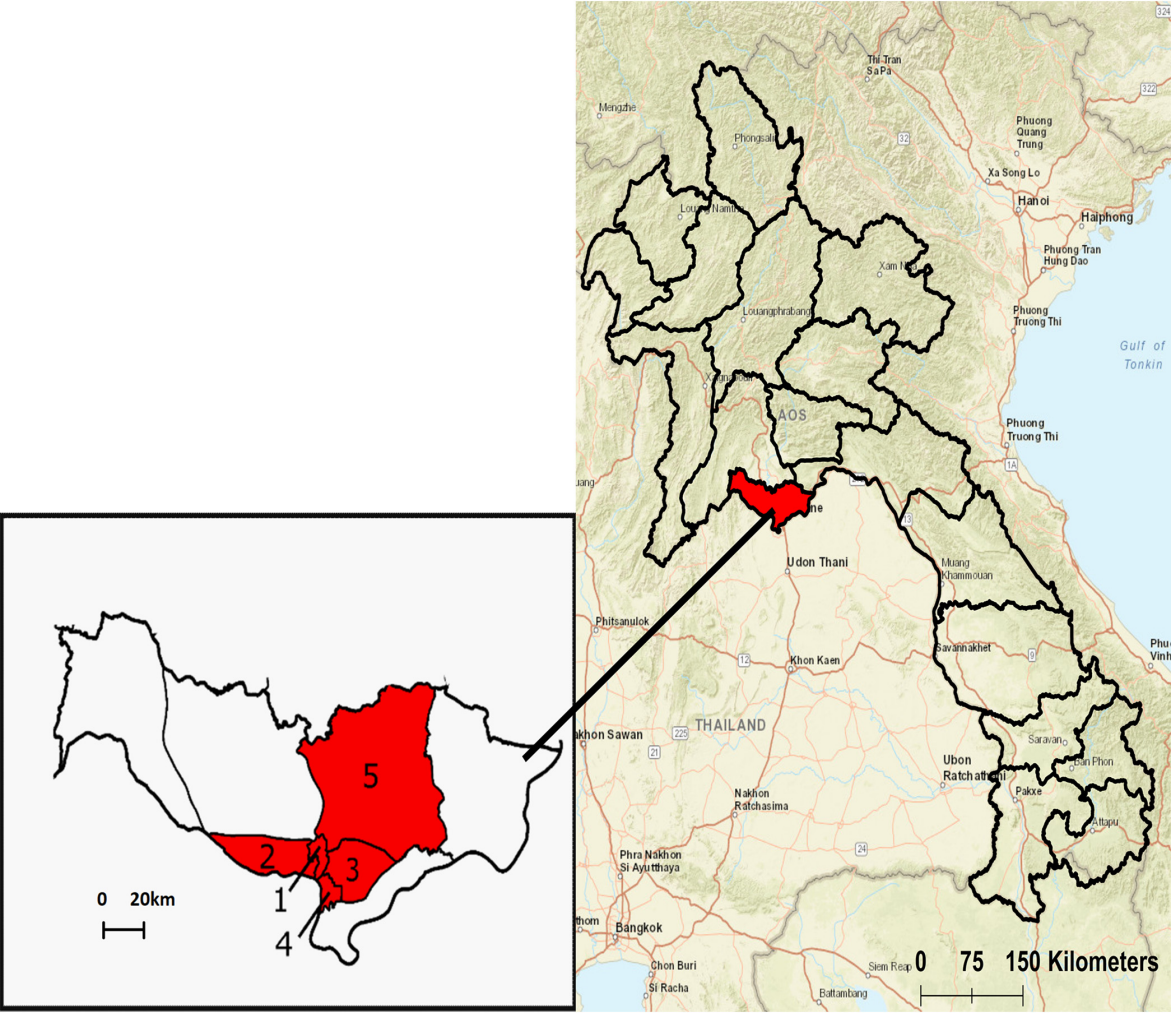
Map of the 18 provinces making up Lao PDR with Vientiane Capital highlighted in red. The expanded view shows Vientiane Capital comprised of its nine districts. Numbers indicate the four urban and one semiurban district where survey sites were located (1, Chanthabuly; 2, Sikhottabong; 3, Xaysetha; 4, Sisattanak; 5, Xathany).

Vientiane Capital is located along the southern edge of Vientiane Plain and is situated on the left bank of Mekong River. The topography is generally flat, with elevations varying no more than 164 to 175 m above sea level (37). Forty drain sites were selected across five urban districts of Vientiane Capital encompassing an area of approximately 100 km^2^: Chanthabuly, Sisattanak, Xaysetha, Xaythany, and Sikhottabong (17.9°N, 102.6°E) (Fig. 1 and 5). Sites were selected based on their accessibility, including proximity to the road and whether they were unfenced and uncovered. The environmental sources of drain water primarily consisted of surface land runoff from stormwater and irrigation and drains varied in their patterns of flow, shading, and lining. Informed oral consent was obtained from landowners and written permission was obtained from the relevant authorities before commencement of sampling.

Samples were collected during the Lao rainy season in late June to July 2018. Eight samples consisting of both soil and water were collected at each of the 40 sites. Three water samples were collected inside each drain line using sterile 1-liter bottles, totaling 120 samples. Five soil samples were also simultaneously collected along adjacent drainage embankment areas (200 samples total), each spaced 10 m apart (5).

On-site physicochemical measurements were analyzed for each sample collected. For water samples, these consisted of nitrate (Horiba LAQUAtwin NO3-11), temperature, pH, electrical conductivity, dissolved oxygen (DO), redox potential (ORP), water turbidity, and total dissolved solids (TDS) using a portable multiparameter field probe (Hanna Instruments; HI9829). Postsampling E. coli and coliform counts were performed on all water samples at the Lao-Oxford-Mahosot Hospital-Wellcome Trust Research Unit (LOMWRU) laboratory in Vientiane Capital (3M Petrifilm). Additionally, drain lining (concrete lined or unlined), degree of shading, and geographical coordinates (Garmin eTrex 30) were recorded at each survey location.

### Sample processing and confirmation.

**(i) Water samples.** Water samples were processed in a biosafety level 2 laboratory (BSL2) facility in the LOMWRU microbiology laboratory. A total of 500 ml of water was filtered in duplicate through 0.2-μm-pore-size, 47-mm-diameter cellulose acetate filters (Merck & Co.) using an electrical pump. To detect B. pseudomallei on water filters and in sediment, we applied two independent methods: conventional culture techniques and PCR after an enrichment step.

*(a) Conventional culture*. One filter was placed in 30 ml of Ashdown broth containing colistin (50 mg/liter) and incubated at 37°C. At 48 h and 7 days postenrichment, 10 μl and 100 μl of broth were plated onto Ashdown's agar with gentamicin (8 mg/liter) and incubated at 37°C aerobically for 2 days. All colonies resembling B. pseudomallei were subcultured onto Ashdown agar. DNA from suspected colonies and from sweeps of bacterial growth on all plates was extracted using 10% Chelex 100 resin (38), and B. pseudomallei was confirmed in-house using a real-time PCR assay targeting a signature 115-bp segment within the bacterial type three secretion system 1 (TTS1) gene (39).

Confirmed B. pseudomallei isolates were stored on tryptone soya broth (TSB) agar slopes in 2-ml screw-cap tubes, incubated at 37°C for 48 h, and stored at room temperature until being shipped to Menzies School of Health Research (Menzies), Darwin, Australia.

*(b) Direct PCR following enrichment*. For direct extraction, one water filter per sample was placed in 30 ml of Ashdown broth containing colistin as described above and shaken at 220 rpm in a 37°C shaking incubator. At 48 h postenrichment, the Ashdown broth was transferred to a sterile 50-ml Falcon tube and spun at 3,400 × *g* for 20 s. The supernatant was transferred to a clean 50-ml Falcon tube and spun at 4,300 × *g* for 45 min. The supernatant was discarded and the pellet was transferred to a 2-ml screw-cap tube and stored at –20°C until being shipped to Menzies for direct extraction and PCR confirmation. Direct extraction from pellets was done using the Qiagen DNeasy PowerSoil DNA isolation kit (Qiagen Pty. Ltd.), and TTS1 qPCR confirmation was performed at Menzies.

**(ii) Soil sample processing.** Technical issues prohibited adequate on-site parallel processing of soil samples to enable valid comparisons between soil and water at study sites and have consequently been excluded from parallel statistical and geospatial analysis. Briefly, soil samples were stored in the dark at room temperature at LOMWRU until January to February 2019. Samples were processed using previously established methods and bacterial growth was subcultured onto TSB agar slopes in 2-ml screw-cap tubes and shipped to Menzies, Darwin, Australia, for culture detection of B. pseudomallei (40–42). DNA was extracted using 10% Chelex 100 resin (38), and B. pseudomallei was confirmed using the TTS1 qPCR assay (39).

### Mapping and statistical analysis.

Maps were created with ArcGIS (version 10.4.1; ESRI, Inc.) using GPS coordinates recorded at sampling sites. The spatial correlation across B. pseudomallei-positive sites was examined in ArcGIS implementing the Global indexes of spatial autocorrelation (Moran’s index) function with a fixed band distance. A positive Moran’s index and Z-score of >1.96 were considered significant at a 95% confidence level (*P* < 0.05) (43).

Statistical analyses were computed with Stata 14.0 (StataCorp.). A semiparametric binomial generalized estimating equation (GEE) model with robust standard errors clustered for site (40 sites) was used to analyze associations between the occurrence of B. pseudomallei and different physicochemical factors by estimating population averaged parameters which are robust to the unknown covariance structure within sites. An exchangeable intraclass correlation coefficient (ICC) was estimated (ICC = 0.04) and odds ratios (ORs) for B. pseudomallei occurrence were calculated. Results were considered significant if *P* values were less than 0.05. Multicollinearity between model predictors was assessed using the variance inflation factor (VIF); all VIF values were less than 1.2. Model residuals were checked, and no patterns across predictors were found.

### Whole-genome sequencing of B. pseudomallei isolates.

Twenty-five Vientiane study isolates were included in the comparative genomic analysis. One B. pseudomallei water isolate from each culture-positive drain site (*n* = 8) was initially selected for WGS. All isolates were chosen at random. To examine genetic variation between sites, additional isolates cultured from all drain samples were screened by BOX-PCR and visualized via gel electrophoresis using methods previously described (44). One isolate was selected from every culture-positive sample within a site (minimum number of isolates per site, 1; maximum number of isolates examined per site, 4) and screened against the single site isolate already selected for WGS. All sample isolates within a site that had banding patterns different from that of the primary WGS isolate were also sent for sequencing (*n* = 13). B. pseudomallei isolates recovered from four positive soil samples at two sites were also included in genomic analysis to increase diversity and phylogenetic resolution. Genomic DNA was extracted using the Qiagen DNeasy blood and tissue kit (Qiagen, Chadstone, Victoria, Australia) as previously described (44). Isolates were sequenced at the Australian Genome Research Facility Ltd. (Melbourne, Australia) using the NovaSeq 6000 platform (Illumina, Inc., San Diego, CA). Genomic analysis included an additional 15 publicly available Lao and 159 global B. pseudomallei genomes, and all genomes are available in the Sequence Read Archive database (Table S1). Read quality was assessed using Trimmomatic v0.39 (45) and FastQC (https://www.bioinformatics.babraham.ac.uk/projects/fastqc), and reads were assembled using the MGAP pipeline (https://github.com/dsarov/MGAP---Microbial-Genome-Assembler-Pipeline/blob/master/MGAP.sh), generating high-quality draft assemblies. Multilocus sequence typing (MLST) assignment of Lao B. pseudomallei environmental soil and water isolates (*n* = 25) was performed with WGS data *in silico* using the Bacterial Isolates Genome Sequence database (BIGSdb) tool accessible on the B. pseudomallei MLST website (http://pubmlst.org/bpseudomallei/) (46).

Orthologous core biallelic single-nucleotide polymorphisms (SNPs) and indels were identified from WGS data using Genome Analysis Toolkit (GATK) in SPANDx v3.2 50, and the closed Thai K96243 genome was used as the reference for all phylogenetic analyses (47, 48). Maximum-parsimony (MP) trees were constructed from core orthologous SNPs and indels using PAUP (v4.0a165) (49) with 1,000 bootstrap replicates. Trees were visualized in FigTree (v1.4.3) (http://tree.bio.ed.ac.uk/software/figtree/) and manipulated using Interactive Tree of Life (iTOL; v4) (https://itol.embl.de) (50).

### Data availability.

Raw sequence data from this study are available in the NCBI database under BioProject number PRJNA659606 and BioSample accession numbers SAMN15920931 to SAMN15920955.

## Supplementary Material

Supplemental file 1

## References

[1] Cheng AC, Currie BJ 2005 Melioidosis: epidemiology, pathophysiology, and management. Clin Microbiol Rev 18:383–416. doi:10.1128/CMR.18.2.383-416.2005. 15831829 PMC1082802

[2] Limmathurotsakul D, Wongratanacheewin S, Teerawattanasook N, Wongsuvan G, Chaisuksant S, Chetchotisakd P, Chaowagul W, Day NP, Peacock SJ 2010 Increasing incidence of human melioidosis in Northeast Thailand. Am J Trop Med Hyg 82:1113–1117. doi:10.4269/ajtmh.2010.10-0038. 20519609 PMC2877420

[3] Phetsouvanh R, Phongmany S, Newton P, Mayxay M, Ramsay A, Wuthiekanun V, White NJ 2001 Melioidosis and Pandora’s box in the Lao People’s Democratic Republic. Clin Infect Dis 32:653–654. doi:10.1086/318713. 11181133

[4] Dance DAB, Luangraj M, Rattanavong S, Sithivong N, Vongnalaysane O, Vongsouvath M, Newton PN 2018 Melioidosis in the Lao People’s Democratic Republic. Trop Med Infect Dis 3:21. doi:10.3390/tropicalmed3010021. PMC6136615 30274419

[5] Limmathurotsakul D, Wuthiekanun V, Chantratita N, Wongsuvan G, Amornchai P, Day NPJ, Peacock SJ 2010 *Burkholderia pseudomallei* is spatially distributed in soil in Northeast Thailand. PLoS Negl Trop Dis 4:e694. doi:10.1371/journal.pntd.0000694. 20532233 PMC2879387

[6] Pearson T, Giffard P, Beckstrom-Sternberg S, Auerbach R, Hornstra H, Tuanyok A, Price EP, Glass MB, Leadem B, Beckstrom-Sternberg JS, Allan GJ, Foster JT, Wagner DM, Okinaka RT, Sim SH, Pearson O, Wu Z, Chang J, Kaul R, Hoffmaster AR, Brettin TS, Robison RA, Mayo M, Gee JE, Tan P, Currie BJ, Keim P 2009 Phylogeographic reconstruction of a bacterial species with high levels of lateral gene transfer. BMC Biol 7:78. doi:10.1186/1741-7007-7-78. 19922616 PMC2784454

[7] McRobb E, Kaestli M, Price EP, Sarovich DS, Mayo M, Warner J, Spratt BG, Currie BJ 2014 Distribution of *Burkholderia pseudomallei* in northern Australia, a land of diversity. Appl Environ Microbiol 80:3463–3468. doi:10.1128/AEM.00128-14. 24657869 PMC4018869

[8] Chewapreecha C, Holden MT, Vehkala M, Valimaki N, Yang Z, Harris SR, Mather AE, Tuanyok A, De Smet B, Le Hello S, Bizet C, Mayo M, Wuthiekanun V, Limmathurotsakul D, Phetsouvanh R, Spratt BG, Corander J, Keim P, Dougan G, Dance DA, Currie BJ, Parkhill J, Peacock SJ 2017 Global and regional dissemination and evolution of *Burkholderia pseudomallei*. Nat Microbiol 2:16263. doi:10.1038/nmicrobiol.2016.263. 28112723 PMC5300093

[9] Price EP, Sarovich DS, Viberg L, Mayo M, Kaestli M, Tuanyok A, Foster JT, Keim P, Pearson T, Currie BJ 2015 Whole-genome sequencing of *Burkholderia pseudomallei* isolates from an unusual melioidosis case identifies a polyclonal infection with the same multilocus sequence type. J Clin Microbiol 53:282–286. doi:10.1128/JCM.02560-14. 25339397 PMC4290970

[10] Sarovich DS, Garin B, De Smet B, Kaestli M, Mayo M, Vandamme P, Jacobs J, Lompo P, Tahita MC, Tinto H, Djaomalaza I, Currie BJ, Price EP 2016 Phylogenomic analysis reveals an Asian origin for African *Burkholderia pseudomallei* and further supports melioidosis endemicity in Africa. mSphere 1:e00089-15. doi:10.1128/mSphere.00089-15. PMC4863585 27303718

[11] De Smet B, Sarovich DS, Price EP, Mayo M, Theobald V, Kham C, Heng S, Thong P, Holden MTG, Parkhill J, Peacock SJ, Spratt BG, Jacobs JA, Vandamme P, Currie BJ 2015 Whole-genome sequencing confirms that *Burkholderia pseudomallei* multilocus sequence types common to both Cambodia and Australia are due to homoplasy. J Clin Microbiol 53:323–326. doi:10.1128/JCM.02574-14. 25392354 PMC4290968

[12] Dale J, Price EP, Hornstra H, Busch JD, Mayo M, Godoy D, Wuthiekanun V, Baker A, Foster JT, Wagner DM, Tuanyok A, Warner J, Spratt BG, Peacock SJ, Currie BJ, Keim P, Pearson T 2011 Epidemiological tracking and population assignment of the non-clonal bacterium, *Burkholderia pseudomallei*. PLoS Negl Trop Dis 5:e1381. doi:10.1371/journal.pntd.0001381. 22180792 PMC3236730

[13] Rachlin A, Mayo M, Webb JR, Kleinecke M, Rigas V, Harrington G, Currie BJ, Kaestli M 2020 Whole-genome sequencing of *Burkholderia pseudomallei* from an urban melioidosis hot spot reveals a fine-scale population structure and localised spatial clustering in the environment. Sci Rep 10:5443. doi:10.1038/s41598-020-62300-8. 32214186 PMC7096523

[14] Ribolzi O, Rochelle-Newall E, Dittrich S, Auda Y, Newton PN, Rattanavong S, Knappik M, Soulileuth B, Sengtaheuanghoung O, Dance DAB, Pierret A 2016 Land use and soil type determine the presence of the pathogen *Burkholderia pseudomallei* in tropical rivers. Environ Sci Pollut Res Int 23:7828–7839. doi:10.1007/s11356-015-5943-z. 26758304 PMC4846699

[15] Zimmermann RE, Ribolzi O, Pierret A, Rattanavong S, Robinson MT, Newton PN, Davong V, Auda Y, Zopfi J, Dance DAB 2018 Rivers as carriers and potential sentinels for *Burkholderia pseudomallei* in Laos. Sci Rep 8:8674. doi:10.1038/s41598-018-26684-y. 29875361 PMC5989208

[16] Baker A, Tahani D, Gardiner C, Bristow KL, Greenhill AR, Warner J 2011 Groundwater seeps facilitate exposure to *Burkholderia pseudomallei*. Appl Environ Microbiol 77:7243–7246. doi:10.1128/AEM.05048-11. 21873480 PMC3194885

[17] Rochelle-Newall E, Nguyen TMH, Le TPQ, Sengtaheuanghoung O, Ribolzi O 2015 A short review of fecal indicator bacteria in tropical aquatic ecosystems: knowledge gaps and future directions. Front Microbiol 6:308. doi:10.3389/fmicb.2015.00308. 25941519 PMC4400915

[18] Chuah CJ, Tan EKH, Sermswan RW, Ziegler AD 2017 Hydrological connectivity and *Burkholderia pseudomallei* prevalence in wetland environments: investigating rice-farming community’s risk of exposure to melioidosis in North-East Thailand. Environ Monit Assess 189:287. doi:10.1007/s10661-017-5988-1. 28536911

[19] Vongphayloth K, Rattanavong S, Moore CE, Phetsouvanh R, Wuthiekanun V, Sengdouangphachanh A, Phouminh P, Newton PN, Buisson Y 2012 *Burkholderia pseudomallei* detection in surface water in southern Laos using Moore’s swabs. Am J Trop Med Hyg 86:872–877. doi:10.4269/ajtmh.2012.11-0739. 22556090 PMC3335696

[20] Limmathurotsakul D, Golding N, Dance DA, Messina JP, Pigott DM, Moyes CL, Rolim DB, Bertherat E, Day NP, Peacock SJ, Hay SI 2016 Predicted global distribution of *Burkholderia pseudomallei* and burden of melioidosis. Nat Microbiol 1:15008. doi:10.1038/nmicrobiol.2015.8. 27571754

[21] Aziz A, Sarovich DS, Harris TM, Kaestli M, McRobb E, Mayo M, Currie BJ, Price EP 2017 Suspected cases of intracontinental *Burkholderia pseudomallei* sequence type homoplasy resolved using whole-genome sequencing. Microb Genom 3:e000139. doi:10.1099/mgen.0.000139. PMC5729916 29208140

[22] Rachlin A, Kleinecke M, Kaestli M, Mayo M, Webb JR, Rigas V, Shilton C, Benedict S, Dyrting K, Currie BJ 2019 A cluster of melioidosis infections in hatchling saltwater crocodiles (*Crocodylus porosus*) resolved using genome-wide comparison of a common north Australian strain of *Burkholderia pseudomallei*. Microb Genom 5:e000288. doi:10.1099/mgen.0.000288. PMC6755496 31433287

[23] Draper AD, Mayo M, Harrington G, Karp D, Yinfoo D, Ward L, Haslem A, Currie BJ, Kaestli M 2010 Association of the melioidosis agent *Burkholderia pseudomallei* with water parameters in rural water supplies in Northern Australia. Appl Environ Microbiol 76:5305–5307. doi:10.1128/AEM.00287-10. 20543039 PMC2916487

[24] Causse J, Billen G, Garnier J, Henri-Des-Tureaux T, Olasa X, Thammahacksa C, Latsachak KO, Soulileuth B, Sengtaheuanghoung O, Rochelle-Newall E, Ribolzi O 2015 Field and modelling studies of *Escherichia coli* loads in tropical streams of montane agro-ecosystems. J Hydro Environment Res 9:496–507. doi:10.1016/j.jher.2015.03.003.

[25] Deiner K, Fronhofer EA, Mächler E, Walser J-C, Altermatt F 2016 Environmental DNA reveals that rivers are conveyer belts of biodiversity information. Nat Commun 7:12544. doi:10.1038/ncomms12544. 27572523 PMC5013555

[26] Gerrard P 2004 Integrating wetland ecosystem values into urban planning: the case of that Luang Marsh. Vientiane, Lao PDR. IUCN—The World Conservation Union Asia Regional Environmental Economics Programme and WWF Lao Country Office, Vientiane, Lao PDR.

[27] Kyophilavong P 2008 Impact of irrigation on aquatic wetland resources: a case study of That Luang Marsh, Lao PDR. EEPSEA, IDRC Regional Office for Southeast and East Asia, Singapore, Singapore.

[28] Knappik M, Dance DAB, Rattanavong S, Pierret A, Ribolzi O, Davong V, Silisouk J, Vongsouvath M, Newton PN, Dittrich S 2015 Evaluation of molecular methods to improve the detection of *Burkholderia pseudomallei* in soil and water samples from Laos. Appl Environ Microbiol 81:3722–3727. doi:10.1128/AEM.04204-14. 25819969 PMC4421066

[29] Kaestli M, Mayo M, Harrington G, Watt F, Hill J, Gal D, Currie BJ 2007 Sensitive and specific molecular detection of *Burkholderia pseudomallei*, the causative agent of melioidosis, in the soil of tropical northern Australia. Appl Environ Microbiol 73:6891–6897. doi:10.1128/AEM.01038-07. 17873073 PMC2074964

[30] Dance D, Knappik M, Dittrich S, Davong V, Silisouk J, Vongsouvath M, Rattanavong S, Pierret A, Newton P, Amornchai P, Wuthiekanun V, Langla S, Limmathurotsakul D 2018 Evaluation of consensus method for the culture of *Burkholderia pseudomallei* in soil samples from Laos. Wellcome Open Res 3:132. doi:10.12688/wellcomeopenres.14851.2. 30569022 PMC6283377

[31] Price EP, Currie BJ, Sarovich DS 2017 Genomic insights into the melioidosis pathogen, *Burkholderia pseudomallei*. Curr Trop Med Rep 4:95–102. doi:10.1007/s40475-017-0111-9.

[32] Chapple SNJ, Price EP, Sarovich DS, McRobb E, Mayo M, Kaestli M, Spratt BG, Currie BJ 2016 *Burkholderia pseudomallei* genotype distribution in the Northern Territory, Australia. Am J Trop Med Hyg 94:68–72. doi:10.4269/ajtmh.15-0627. 26526925 PMC4710447

[33] Hampton V, Kaestli M, Mayo M, Choy JL, Harrington G, Richardson L, Benedict S, Noske R, Garnett ST, Godoy D, Spratt BG, Currie BJ 2011 Melioidosis in birds and *Burkholderia pseudomallei* dispersal, Australia. Emerg Infect Dis 17:1310–1311. doi:10.3201/eid1707.100707. 21762599 PMC3381411

[34] Rattanavong S, Wuthiekanun V, Langla S, Amornchai P, Sirisouk J, Phetsouvanh R, Moore CE, Peacock SJ, Buisson Y, Newton PN 2011 Randomized soil survey of the distribution of *Burkholderia pseudomallei* in rice fields in Laos. Appl Environ Microbiol 77:532–536. doi:10.1128/AEM.01822-10. 21075883 PMC3020526

[35] Wuthiekanun V, Mayxay M, Chierakul W, Phetsouvanh R, Cheng AC, White NJ, Day NPJ, Peacock SJ 2005 Detection of *Burkholderia pseudomallei* in soil within the Lao People’s Democratic Republic. J Clin Microbiol 43:923–924. doi:10.1128/JCM.43.2.923-924.2005. 15695707 PMC548109

[36] Manivanh L, Pierret A, Rattanavong S, Kounnavongsa O, Buisson Y, Elliott I, Maeght JL, Xayyathip K, Silisouk J, Vongsouvath M, Phetsouvanh R, Newton PN, Lacombe G, Ribolzi O, Rochelle-Newall E, Dance DAB 2017 *Burkholderia pseudomallei* in a lowland rice paddy: seasonal changes and influence of soil depth and physico-chemical properties. Sci Rep 7:3031. doi:10.1038/s41598-017-02946-z. 28596557 PMC5465195

[37] Rafiqui PS, Gentile M 2009 Vientiane. Cities 26:38–48. doi:10.1016/j.cities.2008.10.002.

[38] de Lamballerie X, Zandotti C, Vignoli C, Bollet C, de Micco P 1992 A one-step microbial DNA extraction method using “Chelex 100” suitable for gene amplification. Res Microbiol 143:785–790. doi:10.1016/0923-2508(92)90107-y. 1298031

[39] Novak RT, Glass MB, Gee JE, Gal D, Mayo MJ, Currie BJ, Wilkins PP 2006 Development and evaluation of a real-time PCR assay targeting the type III secretion system of *Burkholderia pseudomallei*. J Clin Microbiol 44:85–90. doi:10.1128/JCM.44.1.85-90.2006. 16390953 PMC1351940

[40] Mayo M, Kaesti M, Harrington G, Cheng AC, Ward L, Karp D, Jolly P, Godoy D, Spratt BG, Currie BJ 2011 *Burkholderia pseudomallei* in unchlorinated domestic bore water, Tropical Northern Australia. Emerg Infect Dis 17:1283–1285. doi:10.3201/eid1707.100614. 21762588 PMC3381386

[41] Currie BJ, Price EP, Mayo M, Kaestli M, Theobald V, Harrington I, Harrington G, Sarovich DS 2015 Use of whole-genome sequencing to link *Burkholderia pseudomallei* from air sampling to mediastinal melioidosis, Australia. Emerg Infect Dis 21:2052–2054. doi:10.3201/eid2111.141802. 26488732 PMC4622230

[42] Limmathurotsakul D, Dance DAB, Wuthiekanun V, Kaestli M, Mayo M, Warner J, Wagner DM, Tuanyok A, Wertheim H, Yoke Cheng T, Mukhopadhyay C, Puthucheary S, Day NPJ, Steinmetz I, Currie BJ, Peacock SJ 2013 Systematic review and consensus guidelines for environmental sampling of *Burkholderia pseudomallei*. PLoS Negl Trop Dis 7:e2105. doi:10.1371/journal.pntd.0002105. 23556010 PMC3605150

[43] Waller LA, Gotway CA 2004 Applied spatial statistics for public health data. John Wiley & Sons, Hoboken, NJ.

[44] Currie BJ, Gal D, Mayo M, Ward L, Godoy D, Spratt BG, LiPuma JJ 2007 Using BOX-PCR to exclude a clonal outbreak of melioidosis. BMC Infect Dis 7:68. doi:10.1186/1471-2334-7-68. 17603903 PMC1925088

[45] Bolger AM, Lohse M, Usadel B 2014 Trimmomatic: a flexible trimmer for Illumina sequence data. Bioinformatics 30:2114–2120. doi:10.1093/bioinformatics/btu170. 24695404 PMC4103590

[46] Jolley KA, Maiden MCJ 2010 BIGSdb: scalable analysis of bacterial genome variation at the population level. BMC Bioinformatics 11:595. doi:10.1186/1471-2105-11-595. 21143983 PMC3004885

[47] Sarovich DS, Price EP 2014 SPANDx: a genomics pipeline for comparative analysis of large haploid whole genome re-sequencing datasets. BMC Res Notes 7:618. doi:10.1186/1756-0500-7-618. 25201145 PMC4169827

[48] Holden MT, Titball RW, Peacock SJ, Cerdeno-Tarraga AM, Atkins T, Crossman LC, Pitt T, Churcher C, Mungall K, Bentley SD, Sebaihia M, Thomson NR, Bason N, Beacham IR, Brooks K, Brown KA, Brown NF, Challis GL, Cherevach I, Chillingworth T, Cronin A, Crossett B, Davis P, DeShazer D, Feltwell T, Fraser A, Hance Z, Hauser H, Holroyd S, Jagels K, Keith KE, Maddison M, Moule S, Price C, Quail MA, Rabbinowitsch E, Rutherford K, Sanders M, Simmonds M, Songsivilai S, Stevens K, Tumapa S, Vesaratchavest M, Whitehead S, Yeats C, Barrell BG, Oyston PC, Parkhill J 2004 Genomic plasticity of the causative agent of melioidosis, *Burkholderia pseudomallei*. Proc Natl Acad Sci U S A 101:14240–14245. doi:10.1073/pnas.0403302101. 15377794 PMC521101

[49] Wilgenbusch JC, Swofford D 2003 Inferring evolutionary trees with PAUP*. Curr Protoc Bioinformatics 00:6.4.1–6.4.28. doi:10.1002/0471250953.bi0604s00. 18428704

[50] Letunic I, Bork P 2016 Interactive tree of life (iTOL) v3: an online tool for the display and annotation of phylogenetic and other trees. Nucleic Acids Res 44:W242–W245. doi:10.1093/nar/gkw290. 27095192 PMC4987883

