# Supplementary material for: Using Land Runoff To Survey the Distribution and Genetic Diversity of Burkholderia pseudomallei Strains in Vientiane, Laos
Source: Appl Environ Microbiol. 2021 Jan 29;87(4):e02112-20. doi: 10.1128/AEM.02112-20 (PMC7851688; doi:10.1128/AEM.02112-20)
Supplement: Supplemental file 1 [file AEM.02112-20-s0001.pdf]

Supplementary Data

Table S1- The 199 *B. pseudomallei* genomes used in this study.

***B. pseudomallei* strain**	**Country of Origin**	**Accession no**	**Genome Reference (if applicable)**
MSHR0116	Australia	ERR311040	Chewapreecha et al. Nat Microbiol 2017 (2):16263
MSHR0210	Australia	ERR311043	Chewapreecha et al. Nat Microbiol 2017 (2):16263
MSHR0253	Australia	ERR311045	Chewapreecha et al. Nat Microbiol 2017 (2):16263
MSHR0287	Australia	ERR311046	Chewapreecha et al. Nat Microbiol 2017 (2):16263
MSHR0356	Australia	ERR311049	Chewapreecha et al. Nat Microbiol 2017 (2):16263
MSHR0445a	Australia	ERR311050	Chewapreecha et al. Nat Microbiol 2017 (2):16263
MSHR0447	Australia	ERR311052	Chewapreecha et al. Nat Microbiol 2017 (2):16263
MSHR0668	Australia	SRR1617372	Price EP et al. Curr Trop Med Rep 4:95-102
MSHR0690	Australia	ERR311057	Chewapreecha et al. Nat Microbiol 2017 (2):16263
MSHR0710	Australia	ERR311058	Chewapreecha et al. Nat Microbiol 2017 (2):16263
MSHR0720	Australia	ERR311059	Chewapreecha et al. Nat Microbiol 2017 (2):16263
MSHR0867	Australia	ERR311061	Chewapreecha et al. Nat Microbiol 2017 (2):16263
MSHR0911	Australia	ERR311062	Chewapreecha et al. Nat Microbiol 2017 (2):16263
MSHR1153	Australia	SRR2875414	Johnson et al. Genome Announc. 2015 3(1): e01282-14
MSHR1200	Australia	ERR539740	Chewapreecha et al. Nat Microbiol 2017 (2):16263
MSHR1333	Australia	ERR539742	Chewapreecha et al. Nat Microbiol 2017 (2):16263
MSHR1455	Australia	ERR539744	Chewapreecha et al. Nat Microbiol 2017 (2):16263
MSHR1555	Australia	ERR539745	Chewapreecha et al. Nat Microbiol 2017 (2):16263
MSHR1768	Australia	ERR539750	Chewapreecha et al. Nat Microbiol 2017 (2):16263
MSHR1879	Australia	ERR539751	Chewapreecha et al. Nat Microbiol 2017 (2):16263
MSHR1888	Australia	ERR539752	Chewapreecha et al. Nat Microbiol 2017 (2):16263
MSHR2089	Australia	ERR539755	Chewapreecha et al. Nat Microbiol 2017 (2):16263
MSHR2141	Australia	ERR298341	Chewapreecha et al. Nat Microbiol 2017 (2):16263
MSHR2178	Australia	ERR539756	Chewapreecha et al. Nat Microbiol 2017 (2):16263
MSHR2189	Australia	ERR539757	Chewapreecha et al. Nat Microbiol 2017 (2):16263
MSHR2232	Australia	ERR539758	Chewapreecha et al. Nat Microbiol 2017 (2):16263
MSHR2265	Australia	ERR539759	Chewapreecha et al. Nat Microbiol 2017 (2):16263
MSHR2543	Australia	ERR539761	Chewapreecha et al. Nat Microbiol 2017 (2):16263

MSHR3497	Australia	ERR539764	Chewapreecha et al. Nat Microbiol 2017 (2):16263
MSHR4004	Australia	ERR298343	Chewapreecha et al. Nat Microbiol 2017 (2):16263
MSHR498	Australia	ERR298340	Chewapreecha et al. Nat Microbiol 2017 (2):16263
MSHR504	Australia	ERR298342	Chewapreecha et al. Nat Microbiol 2017 (2):16263
MSHR5477	Australia	ERR298344	Chewapreecha et al. Nat Microbiol 2017 (2):16263
MSHR5619	Australia	ERR298346	Chewapreecha et al. Nat Microbiol 2017 (2):16263
6_CDC	Bangladesh	ERR311033	Chewapreecha et al. Nat Microbiol 2017 (2):16263
H034580128	Bangladesh	ERR298750	Chewapreecha et al. Nat Microbiol 2017 (2):16263
H061220286	Bangladesh	ERR298751	Chewapreecha et al. Nat Microbiol 2017 (2):16263
H103520155	Bangladesh	ERR298752	Chewapreecha et al. Nat Microbiol 2017 (2):16263
SID2889	Bangladesh	ERR298747	Chewapreecha et al. Nat Microbiol 2017 (2):16263
SID3811	Bangladesh	ERR298748	Chewapreecha et al. Nat Microbiol 2017 (2):16263
SID4717	Bangladesh	ERR298749	Chewapreecha et al. Nat Microbiol 2017 (2):16263
4900CFPatient1	Brazil	ARZE00000000	Gee et al., Emerg Infect Dis 2014 20(4):682-84
BCC215	Brazil	ABBR00000000	Gee et al., Emerg Infect Dis 2014 20(4):682-84
H061740680	Brazil	ERR298754	Chewapreecha et al. Nat Microbiol 2017 (2):16263
SID1615	Brazil	ERR298753	Chewapreecha et al. Nat Microbiol 2017 (2):16263
SID1131	Brunei	ERR298755	Chewapreecha et al. Nat Microbiol 2017 (2):16263
BF103; MSHR7964	Burkina Faso	SRR3145394	Sarovich et al mSphere 2016 1(2): e00089-15
BF111; MSHR7965	Burkina Faso	SRR3145395	Sarovich et al mSphere 2016 1(2): e00089-15
NT_20	Burkina_Faso	ERR298756	Chewapreecha et al. Nat Microbiol 2017 (2):16263
CAM41	Cambodia	ERR539773	Chewapreecha et al. Nat Microbiol 2017 (2):16263
E562	Cambodia	ERR178253	Chewapreecha et al. Nat Microbiol 2017 (2):16263
E569	Cambodia	ERR178254	Chewapreecha et al. Nat Microbiol 2017 (2):16263
SHCH2430	Cambodia	ERR298360	Chewapreecha et al. Nat Microbiol 2017 (2):16263
SR-020	Cambodia	ERR178250	Chewapreecha et al. Nat Microbiol 2017 (2):16263
SR-025	Cambodia	ERR178251	Chewapreecha et al. Nat Microbiol 2017 (2):16263
SR-039	Cambodia	ERR178245	Chewapreecha et al. Nat Microbiol 2017 (2):16263
5691	Chad	ERR298779	Chewapreecha et al. Nat Microbiol 2017 (2):16263
MSHR6969	Chad	ERR298347	Chewapreecha et al. Nat Microbiol 2017 (2):16263
BPC006	China	NC_018527, NC_018529	Fang et al., 2012 J Bacteriol 194(23) 6604-05
E0698	China	ERR539770	Chewapreecha et al. Nat Microbiol 2017 (2):16263
E0702	China	ERR539771	Chewapreecha et al. Nat Microbiol 2017 (2):16263

Hainan_106	China	ERR298758	Chewapreecha et al. Nat Microbiol 2017 (2):16263
Hainan1	China	ERR298757	Chewapreecha et al. Nat Microbiol 2017 (2):16263
HK2	China	ERR539766	Chewapreecha et al. Nat Microbiol 2017 (2):16263
OP285A	China	ERR539769	Chewapreecha et al. Nat Microbiol 2017 (2):16263
OP79A	China	ERR539767	Chewapreecha et al. Nat Microbiol 2017 (2):16263
7893	Ecuador	ERR351982	Chewapreecha et al. Nat Microbiol 2017 (2):16263
MSHR0142	Fiji	ERR311042	Chewapreecha et al. Nat Microbiol 2017 (2):16263
MSHR3020	Fiji	ERR539763	Chewapreecha et al. Nat Microbiol 2017 (2):16263
C2_Bp	Gabon	ERR403714	Chewapreecha et al. Nat Microbiol 2017 (2):16263
Gabon_Bp	Gabon	ERR403716	Chewapreecha et al. Nat Microbiol 2017 (2):16263
G9709	India	ERR311037	Chewapreecha et al. Nat Microbiol 2017 (2):16263
H054640145	India	ERR298759	Chewapreecha et al. Nat Microbiol 2017 (2):16263
H103360117	India	ERR298760	Chewapreecha et al. Nat Microbiol 2017 (2):16263
MSHR2056	Indonesia	ERR539754	Chewapreecha et al. Nat Microbiol 2017 (2):16263
MM24	Laos	ERR162597	Chewapreecha et al. Nat Microbiol 2017 (2):16263
MM28	Laos	ERR162604	Chewapreecha et al. Nat Microbiol 2017 (2):16263
MM37	Laos	ERR162603	Chewapreecha et al. Nat Microbiol 2017 (2):16263
MM39	Laos	ERR162598	Chewapreecha et al. Nat Microbiol 2017 (2):16263
MM45	Laos	ERR162599	Chewapreecha et al. Nat Microbiol 2017 (2):16263
MM54	Laos	ERR162601	Chewapreecha et al. Nat Microbiol 2017 (2):16263
MM56	Laos	ERR162605	Chewapreecha et al. Nat Microbiol 2017 (2):16263
MM70	Laos	ERR162607	Chewapreecha et al. Nat Microbiol 2017 (2):16263
MM76	Laos	ERR162606	Chewapreecha et al. Nat Microbiol 2017 (2):16263
MM80	Laos	ERR162608	Chewapreecha et al. Nat Microbiol 2017 (2):16263
MM85	Laos	ERR162600	Chewapreecha et al. Nat Microbiol 2017 (2):16263
MM91	Laos	ERR162602	Chewapreecha et al. Nat Microbiol 2017 (2):16263
SVC_34	Laos	ERR162611	Chewapreecha et al. Nat Microbiol 2017 (2):16263
SVE_77	Laos	ERR162609	Chewapreecha et al. Nat Microbiol 2017 (2):16263
SVW_02	Laos	ERR162610	Chewapreecha et al. Nat Microbiol 2017 (2):16263
MSHR11966	Laos	SAMN15920931	This study
MSHR12103	Laos	SAMN15920932	This study
MSHR12336	Laos	SAMN15920933	This study
MSHR12338	Laos	SAMN15920934	This study

MSHR12369	Laos	SAMN15920935	This study
MSHR12077	Laos	SAMN15920936	This study
MSHR12020	Laos	SAMN15920937	This study
MSHR12347	Laos	SAMN15920938	This study
MSHR12414	Laos	SAMN15920939	This study
MSHR12000	Laos	SAMN15920940	This study
MSHR12071	Laos	SAMN15920941	This study
MSHR11998	Laos	SAMN15920942	This study
MSHR11836	Laos	SAMN15920943	This study
MSHR12048	Laos	SAMN15920944	This study
MSHR12046	Laos	SAMN15920945	This study
MSHR11846	Laos	SAMN15920946	This study
MSHR12097	Laos	SAMN15920947	This study
MSHR12122	Laos	SAMN15920948	This study
MSHR11848	Laos	SAMN15920949	This study
MSHR12054	Laos	SAMN15920950	This study
MSHR12059	Laos	SAMN15920951	This study
MSHR11855	Laos	SAMN15920952	This study
MSHR12061	Laos	SAMN15920953	This study
MSHR11859	Laos	SAMN15920954	This study
MSHR12012	Laos	SAMN15920955	This study
MSHR7966	Madagascar	SRR3145396	Sarovich et al. mSphere 2016 1(2): e00089-15
LN_10	Malaysia	ERR298768	Chewapreecha et al. Nat Microbiol 2017 (2):16263
LN_22892	Malaysia	ERR298767	Chewapreecha et al. Nat Microbiol 2017 (2):16263
LN_29564	Malaysia	ERR298764	Chewapreecha et al. Nat Microbiol 2017 (2):16263
LN_31348	Malaysia	ERR298765	Chewapreecha et al. Nat Microbiol 2017 (2):16263
LN_34170	Malaysia	ERR298763	Chewapreecha et al. Nat Microbiol 2017 (2):16263
LN_34677	Malaysia	ERR298769	Chewapreecha et al. Nat Microbiol 2017 (2):16263
LN_6	Malaysia	ERR298766	Chewapreecha et al. Nat Microbiol 2017 (2):16263
MSHR0315	Malaysia	ERR311047	Chewapreecha et al. Nat Microbiol 2017 (2):16263
SID3783	Malaysia	ERR298771	Chewapreecha et al. Nat Microbiol 2017 (2):16263
SID4349	Malaysia	ERR298770	Chewapreecha et al. Nat Microbiol 2017 (2):16263
MSHR7398	Martinique	ERR298357	Chewapreecha et al. Nat Microbiol 2017 (2):16263

MSHR7400	Martinique	ERR298359	Chewapreecha et al. Nat Microbiol 2017 (2):16263
Green	Mauritius	ERR311038	Chewapreecha et al. Nat Microbiol 2017 (2):16263
IL2014_Mexico	Mexico	SRR7140691	Donahue et al. Open Forum Infect Dis. 2019 6(2):ofz005
MSHR7397	New_Caledonia	ERR298356	Chewapreecha et al. Nat Microbiol 2017 (2):16263
MSHR7399	New_Caledonia	ERR298358	Chewapreecha et al. Nat Microbiol 2017 (2):16263
H101180656	Nigeria	ERR298772	Chewapreecha et al. Nat Microbiol 2017 (2):16263
MSHR0491	Northern Territory, Australia	ERR311054	Chewapreecha et al. Nat Microbiol 2017 (2):16263
Pakistan9	Pakistan	CP008754, CP008755	Daligault et al., Genome Announc 2014 2(6):e01106-14
G1467	Papua New Guinea	ERR311036	Chewapreecha et al. Nat Microbiol 2017 (2):16263
MSHR0141	Papua New Guinea	ERR311041	Chewapreecha et al. Nat Microbiol 2017 (2):16263
MSHR2434	Papua New Guinea	ERR539760	Chewapreecha et al. Nat Microbiol 2017 (2):16263
H110440887	Philippines	ERR298777	Chewapreecha et al. Nat Microbiol 2017 (2):16263
MK_1900	Philippines	ERR298776	Chewapreecha et al. Nat Microbiol 2017 (2):16263
MK_441	Philippines	ERR298773	Chewapreecha et al. Nat Microbiol 2017 (2):16263
MK_451	Philippines	ERR298774	Chewapreecha et al. Nat Microbiol 2017 (2):16263
MK_453	Philippines	ERR298775	Chewapreecha et al. Nat Microbiol 2017 (2):16263
F3253	Puerto_Rico	ERR311035	Chewapreecha et al. Nat Microbiol 2017 (2):16263
H0929	Puerto_Rico	ERR311039	Chewapreecha et al. Nat Microbiol 2017 (2):16263
MSHR6976	Sabah(Malaysia)	ERR298354	Chewapreecha et al. Nat Microbiol 2017 (2):16263
MSHR5107	Sarawak, Malaysian Borneo	JZXP00000000	Podin et al., 2014 Antimicob Agents Chemother 58 (1), 162-6
Bp22	Singapore	CM001156, CM001157	Nandi et al., 2010 PLoS Pathog 6(4): E1000845
MSHR6975	Singapore	ERR298353	Chewapreecha et al. Nat Microbiol 2017 (2):16263
S13	Singapore	AAHW02000000	Nandi et al., 2010 PLoS Pathog 6(4): E1000845
1426a	Thailand	ERR162622	Chewapreecha et al. Nat Microbiol 2017 (2):16263
2614a	Thailand	ERR162612	Chewapreecha et al. Nat Microbiol 2017 (2):16263
2632a	Thailand	ERR162625	Chewapreecha et al. Nat Microbiol 2017 (2):16263
2659a	Thailand	ERR162614	Chewapreecha et al. Nat Microbiol 2017 (2):16263
2667a	Thailand	ERR162615	Chewapreecha et al. Nat Microbiol 2017 (2):16263
2698a	Thailand	ERR162620	Chewapreecha et al. Nat Microbiol 2017 (2):16263
2719a	Thailand	ERR162619	Chewapreecha et al. Nat Microbiol 2017 (2):16263
2767a	Thailand	ERR162613	Chewapreecha et al. Nat Microbiol 2017 (2):16263
2769a	Thailand	ERR162621	Chewapreecha et al. Nat Microbiol 2017 (2):16263
2774a	Thailand	ERR162618	Chewapreecha et al. Nat Microbiol 2017 (2):16263

2820a	Thailand	ERR162617	Chewapreecha et al. Nat Microbiol 2017 (2):16263
2833a	Thailand	ERR162624	Chewapreecha et al. Nat Microbiol 2017 (2):16263
995a	Thailand	ERR162623	Chewapreecha et al. Nat Microbiol 2017 (2):16263
A-277-02-2-10	Thailand	ERR343795	Chewapreecha et al. Nat Microbiol 2017 (2):16263
A-278-05-2-01	Thailand	ERR343796	Chewapreecha et al. Nat Microbiol 2017 (2):16263
A-281-02-2-01	Thailand	ERR343806	Chewapreecha et al. Nat Microbiol 2017 (2):16263
A-281-02-2-02	Thailand	ERR343807	Chewapreecha et al. Nat Microbiol 2017 (2):16263
A-281-02-2-05	Thailand	ERR343810	Chewapreecha et al. Nat Microbiol 2017 (2):16263
A-282-02-1-02	Thailand	ERR343816	Chewapreecha et al. Nat Microbiol 2017 (2):16263
A-294-05-2-03	Thailand	ERR343837	Chewapreecha et al. Nat Microbiol 2017 (2):16263
E361	Thailand	ERR162626	Chewapreecha et al. Nat Microbiol 2017 (2):16263
HBPUB10134a	Thailand	CP008911, CP008912	Daligault et al., Genome Announc 2014 2(6):e01106-14
HBPUB10303a	Thailand	CP008893, CP008894	Daligault et al., Genome Announc 2014 2(6):e01106-14
K96243	Thailand	NC_006350, NC_006351	Holden et al., Proc Natl Acad Sci USA 2004 101(39):14240-45
1106a	Ubon Ratchathani, Thailand	CP008758, CP008759	Daligault et al., Genome Announc 2014 2(6):e01106-14
1106b	Ubon Ratchathani, Thailand	CM000774, CM000775	Nandi et al., 2010 PLoS Pathog 6(4): E1000845
1710a	Ubon Ratchathani, Thailand	CM000832, CM000833	Nandi et al., 2010 PLoS Pathog 6(4): E1000845
1710b	Ubon Ratchathani, Thailand	CP000124, CP000125	Nandi et al., 2010 PLoS Pathog 6(4): E1000845
D4899	Venezuela	ERR351981	Chewapreecha et al. Nat Microbiol 2017 (2):16263
137	Vietnam	ERR178256	Chewapreecha et al. Nat Microbiol 2017 (2):16263
532	Vietnam	ERR178258	Chewapreecha et al. Nat Microbiol 2017 (2):16263
683	Vietnam	ERR178259	Chewapreecha et al. Nat Microbiol 2017 (2):16263
720	Vietnam	ERR178260	Chewapreecha et al. Nat Microbiol 2017 (2):16263
1040	Vietnam	ERR178261	Chewapreecha et al. Nat Microbiol 2017 (2):16263
1042	Vietnam	ERR178262	Chewapreecha et al. Nat Microbiol 2017 (2):16263
1043	Vietnam	ERR178263	Chewapreecha et al. Nat Microbiol 2017 (2):16263
1062	Vietnam	ERR178264	Chewapreecha et al. Nat Microbiol 2017 (2):16263
1072	Vietnam	ERR178265	Chewapreecha et al. Nat Microbiol 2017 (2):16263
1080	Vietnam	ERR178266	Chewapreecha et al. Nat Microbiol 2017 (2):16263
1095	Vietnam	ERR178267	Chewapreecha et al. Nat Microbiol 2017 (2):16263
1097	Vietnam	ERR178268	Chewapreecha et al. Nat Microbiol 2017 (2):16263
1258	Vietnam	ERR178269	Chewapreecha et al. Nat Microbiol 2017 (2):16263
MSHR6970	Vietnam	ERR298348	Chewapreecha et al. Nat Microbiol 2017 (2):16263

MSHR6971	Vietnam	ERR298349	Chewapreecha et al. Nat Microbiol 2017 (2):16263
MSHR6972	Vietnam	ERR298350	Chewapreecha et al. Nat Microbiol 2017 (2):16263
MSHR6973	Vietnam	ERR298351	Chewapreecha et al. Nat Microbiol 2017 (2):16263
MSHR6974	Vietnam	ERR298352	Chewapreecha et al. Nat Microbiol 2017 (2):16263
H065460522	Virgin Islands	ERR298778	Chewapreecha et al. Nat Microbiol 2017 (2):16263
MSHR8441	Thailand	NZ_JYBK00000000	Viberg et al., Genome Announc 2015 3(2):e00254-15

Table S2- Median physico-chemical water parameters, drain descriptions and detection of *B. pseudomallei* in water samples at each of the 40 sites.

Site ID	District	pH	EC (µS/cm)	Temp (C°)	Nitrate (mg/L)	ORP (mV)	TDS (ppm)	Turbidity (FNU)	DO (mg/L)	*E. coli* (CFU/ml)	Coliforms (CFU/ml)	Cement lined	Shaded	*B. pseudomallei* detected in water
1	Sisattanak District	7.3	89	29.2	11	125.5	244	121	1.2	0	0	No	No	Yes
2	Sisattanak District	7.3	411	29.1	14	-109.5	106	18.3	1.8	0	5	Yes	No	No
3	Sisattanak District	7.4	540	29	16	45.3	69	14.6	0.8	1	12	No	No	Yes
4	Sisattanak District	7.04	431	28.3	14	-57.2	115	29.1	0	0	5	No	Yes	Yes
5	Sisattanak District	7	387	27	17	136.6	192	267	3.2	0	3	No	Yes	Yes
6	Sisattanak District	7.1	245	26.3	12	28.4	123	57.9	2.5	0	9	No	No	Yes
7	Sisattanak District	6.6	345	27.4	16	-5.2	173	40.5	3	0	3	No	No	Yes
8	Sisattanak District	6.8	338	28.6	12	36	169	25.2	2.2	0	3	No	No	Yes
9	Xaysetha District	7.1	406	28.2	13	10.2	203	26.3	1.2	0	60	No	No	Yes
10	Sisattanak District	6.8	500	26.9	11	-78.1	249	105	0.5	1	27	Yes	No	Yes
11	Sisattanak District	6.7	551	27.6	14	-59.6	276	80.5	0.9	0	5	No	Yes	Yes
12	Chantabuly District	8.1	290	27.9	11	95.1	145	9.5	0	0	1	Yes	Yes	No
13	Sisattanak District	7.3	412	28.9	16	-52.6	215	115.2	2.4	0	3	No	Yes	No
14	Sisattanak District	6.8	298	30.6	17	30.1	149	143	3.2	0	0	Yes	Yes	Yes
15	Xaysetha District	7	465	27.4	23	-33.3	132	69.2	0.6	0	0	No	No	Yes
16	Xaysetha District	7.2	397	28.1	11	-56.7	198	123	2.2	6	60	No	No	Yes
17	Chanthabuly District	7.4	566	29.3	16	-95.2	85	88	2.8	0	2	No	No	No
18	Xaysetha District	7	174	27.6	11	1.9	287	72.3	1.4	0	11	No	Yes	Yes
19	Xaysetha District	7.3	487	28.3	15	-58.5	249	48.6	1.3	4	24	No	No	Yes
20	Xaysetha District	7.1	386	27.8	11	106.6	180	13.2	3.1	0	8	No	Yes	Yes
21	Xaythany District	7.1	251	29.3	13	-134.9	146	34.5	3.2	1	28	No	No	Yes
22	Xaysetha District	6.9	276	29.8	12	-13	135	131.2	3.5	0	2	No	No	Yes
23	Xaythany District	6.8	297	29.8	11	-50.7	152	32.6	3	0	2	No	No	Yes
24	Xaythany District	6.8	171	29.7	14	-14.2	181	42.3	2.4	0	1	No	Yes	Yes
25	Sikhottabong District	7.2	856	29.8	16	-81.6	198	92	0.5	140	63	No	No	Yes

26	Chanthabuly District	7.2	566	31.6	18	-81.2	212	10	3.7	2	29	No	No	Yes
27	Chanthabuly District	7.1	252	31.2	15	42.5	136	39.4	3.7	0	17	No	No	Yes
28	Sikhottabong District	6.9	396	31.8	19	-73.4	121	59.5	4.1	9	29	No	No	No
29	Sikhottabong District	7.2	418	30.7	13	-77.5	209	13.8	4	8	40	Yes	Yes	No
30	Sikhottabong District	7.2	232	30.4	10	-34	116	31.6	2	0	0	No	No	No
31	Sikhottabong District	6.9	84	30.8	10	117.3	42	24.2	1.9	0	0	No	No	No
32	Sikhottabong District	6.6	155	29.6	8	52.1	78	33.8	2.5	0	7	Yes	No	No
33	Chanthabuly District	7.3	532	29.4	15	-75.3	165	34.8	2	14	57	No	No	Yes
34	Sikhottabong District	7.3	549	29.7	15	-67.2	274	41.8	1.7	92	180	No	Yes	No
35	Chanthabuly District	7	372	29.3	13	-80.7	186	13	0.7	2	73	Yes	Yes	No
36	Chanthabuly District	7.1	384	31	13	-64	191	100	0.9	1	5	No	No	No
37	Xaythany District	7.2	305	29.4	14	71.2	153	32.7	1.2	1	32	No	No	Yes
38	Chanthabuly District	7.1	228	31.2	12	110	114	27.5	1.3	0	31	Yes	No	No
39	Xaythany District	6.8	398	30.4	14	-52.3	116	80	1.5	0	7	No	Yes	Yes
40	Chanthabuly District	7.3	543	31.1	15	-53.5	171	28.9	1.8	7	23	Yes	Yes	No

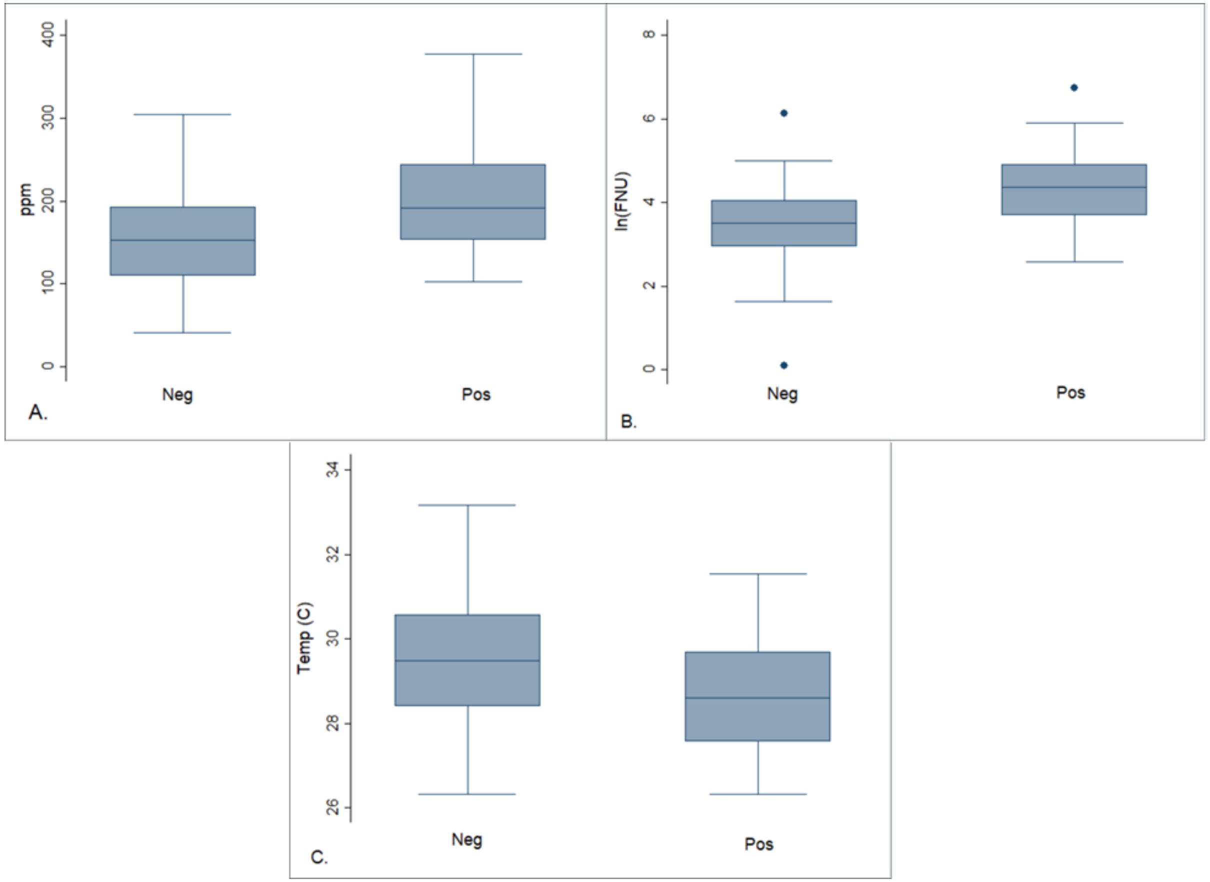

Figure S1- Box-and-whisker plots of significant univariable physico-chemical parameters from *B. pseudomallei*-negative (Neg) and positive (Pos) samples using a GEE model clustered by site. Box plots span the interquartile range of the data with the median marked by a vertical line. Outliers are denoted by solid blue circles**.** (**3A**) TDS (ppm) from positive and negative samples (p=0.002), (**3B**) turbidity (log transformed ln(FNU)) (p=<0.001), (**3C**) water temperature (°C) (p=0.005).

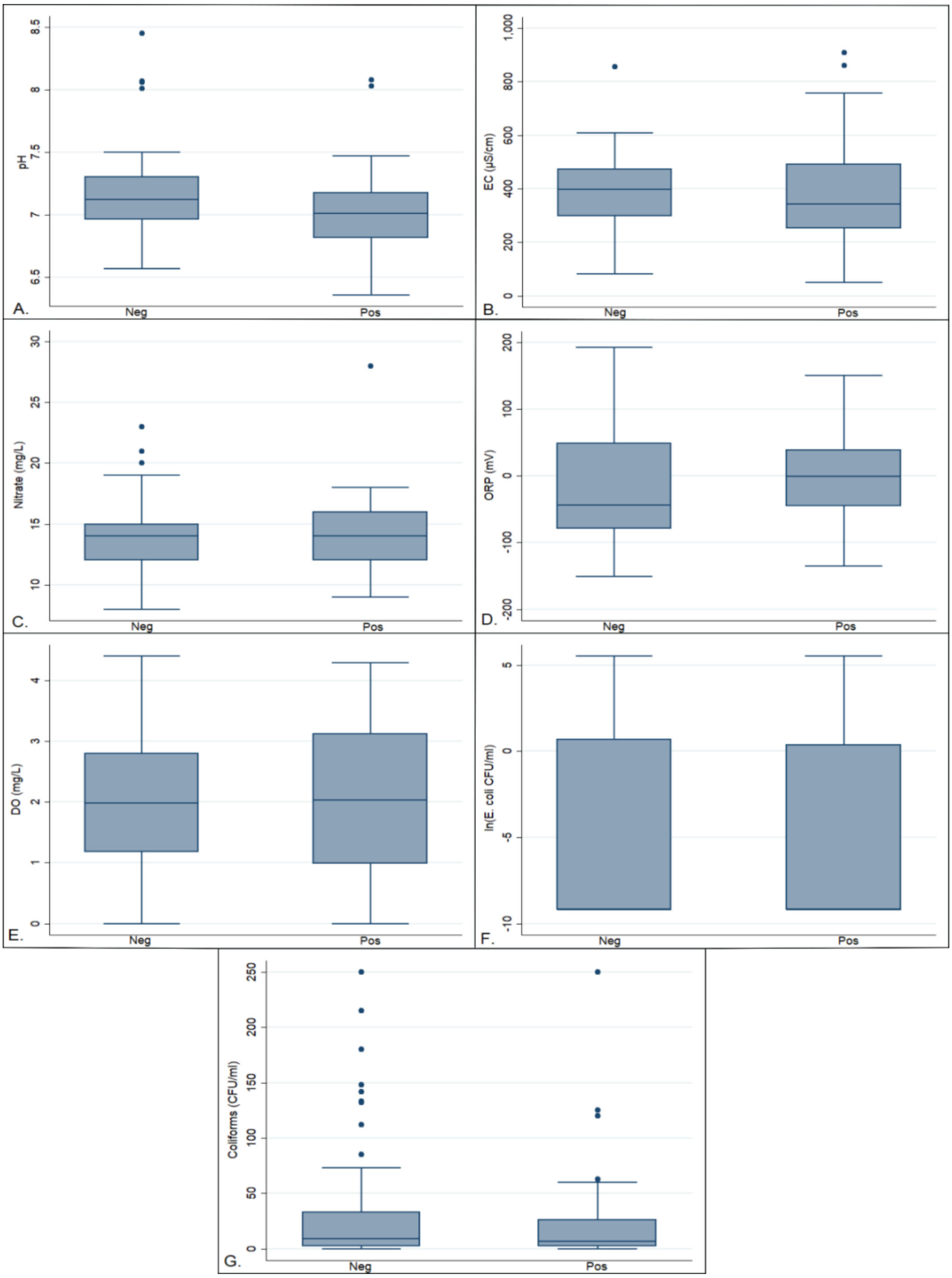

Figure S2- Box-and-whisker plots of non-significant (p>0.05) univariable water parameters from *B. pseudomallei*-negative (Neg) and positive (Pos) samples using a GEE model clustered by site. Box plots span the interquartile range of the data with the median marked by a vertical line. Outliers are denoted by solid blue circles**.** Box plots: pH **(A.),** EC **(B.)**, Nitrate **(C.)**, ORP **(D.)**, DO **(E.)**, *E. coli* **(F.)**, and coliforms **(G.)**.
